# *Caenorhabditis elegans* in neuroscience: from neural communication to neurodegenerative disease modeling

**DOI:** 10.3389/fnagi.2026.1865186

**Published:** 2026-07-20

**Authors:** Ziheng Pan, Yunlong Shen, Dejian Peng, Li Tang, Tianlu Ran, Yaohui Liu, Xinyi Zeng, Hui Liu

**Affiliations:** Health Science Center, Yangtze University, Jingzhou, China

**Keywords:** Alzheimer’s disease, *Caenorhabditis elegans*, model organism, neurodegenerative disease, synaptic transmission

## Abstract

Consistent with humans and other metazoans, the nematode *Caenorhabditis elegans* (*C. elegans*) undergoes progressive structural and functional decline during aging. Possessing highly conserved genetic pathways that share extensive homology with human genes, and characterized by a streamlined, fully mapped connectome, *C. elegans* has emerged as a robust model for dissecting the mechanisms underlying neuronal aging and degeneration. In this review, we summarize the intrinsic advantages of *C. elegans* as a model organism, highlighting its readily quantifiable behavioral phenotypes, short lifespan, and genetic tractability. We elaborate on its foundational neural communication architecture and its unique utility in constructing molecular models of neurodegenerative diseases. Additionally, we explore the integration of this model system with high-throughput pharmacological screening, environmental toxicology evaluations, and advanced genomic sequencing technologies. Ultimately, this synthesis aims to provide a comprehensive framework for investigating neurodegenerative mechanisms and facilitating clinical translation under specific stress conditions, particularly hypoxia.

## Graphical abstract

**Figure:**
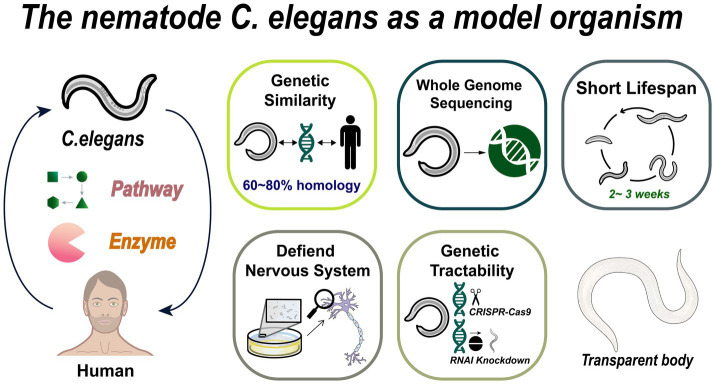

## 1 Introduction

The primary objective of neuroscience is to elucidate how the nervous system enables organisms to execute complex functions, ranging from sensory perception and homeostasis to advanced cognitive behaviors. This neural adaptability allows diverse biological species to thrive in volatile environments (Cell editorial team, 2024). Contemporary neurobiology is undergoing a significant shift toward resolving dynamic, subcellular processes within living mammals. This advancement builds directly upon foundational, in-depth studies originally conducted in invertebrates and optically transparent vertebrates (Jan, 2018). The non-parasitic nematode *Caenorhabditis elegans* (*C. elegans*) serves as a premier model organism in this context. Since its introduction as an experimental model in the 1970s, this millimeter-sized organism has offered unique and enduring research value. It is characterized by a minute body size, self-fertilization capacity, a brief life cycle, and low maintenance costs; at 20 °C, it matures into an adult within just three days. These biological traits, combined with accessible forward genetic screening and straightforward CRISPR-Cas9 genome editing, render *C. elegans* an ideal platform for high-throughput drug screening and dissecting the molecular mechanisms of complex phenomena such as aging (Brenner, 1974; Liang et al., 2024). Wild-type *C. elegans* populations primarily comprise self-fertilizing hermaphrodites, with males naturally occurring at a frequency below 0.2%. This reproductive strategy, along with a large brood size, significantly streamlines the generation of novel lines through genetic crosses (Salinas and Risi, 2018). Consequently, the research utility of this nematode extends across diverse biomedical domains, ranging from ontogeny and aging to pathological modeling, toxicology, and pharmacological screening, establishing it as a foundational model in modern biology.

In nature, *C. elegans* primarily thrives in microbe-rich, decaying organic matter. In contrast, standard laboratory protocols utilize *Escherichia coli* as the primary food source, maintaining the nematodes on Nematode Growth Medium agar plates. The life cycle of *C. elegans* encompasses an embryonic phase, four sequential larval stages (L1 to L4), and a final reproductive adult stage (Zhang et al., 2020). Newly hatched L1 larvae immediately arrest development upon nutrient deprivation. When subjected to starvation, crowding, or thermal stress, L2-stage larvae bypass the standard reproductive cycle to enter the dauer stage—a highly stress-resistant, dormant state. Similarly, egg-laying adults experiencing sudden nutrient deprivation halt germline stem cell division and may even resorb existing embryos to prioritize maternal survival, resuming reproduction only when food availability is restored (Blaxter and Denver, 2012; Corsi et al., 2015). As the first multicellular organism to achieve complete genome sequencing, *C. elegans* serves as a foundational model for human disease due to its genetic conservation.

Sequence alignments indicate that roughly 60 to 80% of human protein-coding genes share recognizable homologs with the nematode (Culetto and Sattelle, 2000; Rubin et al., 2000), despite the vast structural differences in the non-coding regions that constitute 98% of the human genome. This conservation of functional coding networks allows for robust modeling of various neurodegenerative pathways, which is heavily facilitated by the vast strain repository available through the Caenorhabditis Genetics Center (Harris et al., 2004; Torres et al., 2025; Pir et al., 2026). Despite its structural simplicity, *C. elegans* exhibits complex behaviors such as navigation, learning, and decision-making, all coordinated by compact neural circuits. Anatomically, the 302 total neurons are partitioned into a 20-cell pharyngeal system and a 282-cell somatic system. Excluding three specific neurons (CANL/R and VC06) due to their unique connectivity profiles, the remaining 279 core somatic neurons constitute the standard platform for network analysis (White et al., 1986; Nicosia et al., 2013). Although synaptic counts are subject to minor variance across different reconstructions, Varshney et al. (2011) remains the widely cited compilation standard. Under this framework, the core somatic network encompasses 6,393 chemical synapses, 890 gap junctions, and 1,410 neuromuscular junctions (Lapierre and Hansen, 2012). Genetic manipulation in *C. elegans* is remarkably facile. Mutations are typically induced in hermaphrodites through targeted genetic methods, and these alterations are subsequently inherited by offspring via self-fertilization, thereby bypassing the requirement for mating (Liu et al., 2026). Compared to mammals, this nematode offers a fully resolved cell lineage and an exceptionally precise synaptic network, making it an excellent model for studying human pathologies (Eck et al., 2023).

This review systematically articulates the biological characteristics of *C. elegans* and its unique advantages in behavioral neuroscience. As a powerful model organism, this nematode facilitates precise quantitative measurements and in-depth mechanistic dissections of complex neural behaviors and disease phenotypes. To bridge the gap left by previous reviews that often overlook model constraints, this article systematically compares design choices with a particular emphasis on the explicit drawbacks of each *C. elegans* model of neurodegenerative diseases, thereby providing a more rigorous framework for evaluating their interpretability and clinical relevance.

## 2 The nervous system structure of *C. elegans*

*C. elegans* offers a highly advantageous whole-organism system for exploring the pathogenic mechanisms of human neurological diseases. This suitability is largely attributed to the fact that *C. elegans* possesses orthologs for nearly half of all human disease-associated genes; furthermore, its fundamental neurobiological processes, such as synaptic transmission and neural development, remain highly conserved across evolution. Facilitated by a completely sequenced genome and a well-defined neural connectome map, this nematode has become an essential model organism in contemporary neuroscientific research (Roussos et al., 2023).

### 2.1 Synaptic organization and connectivity

Organisms rely on the nervous system to process internal and external stimuli, generating adaptive physiological responses. Determining the specific contributions of individual neurons or distinct brain regions to these mechanisms remains a central objective in neuroscience (Cisek and Green, 2024). Synapses are specialized structures mediating communication between neurons and target cells. Despite hundreds of millions of years of evolution, their molecular composition and functional architecture remain remarkably consequently, the fundamental principles of neural activity can be effectively deciphered by examining the synaptic networks of anatomically tractable organisms, such as *C. elegans* (Emmons, 2024).

In a landmark study, White et al. extensively elucidated the nervous system of *C. elegans* using serial section electron microscopy, reconstructing the spatial connectivity of its 302 neurons. This structural foundation has enabled researchers to visualize broader functional synaptic architectures in live animals via calcium imaging and fluorescence intensity quantification (White et al., 1986; Frankel and Kurshan, 2025). During development, neurons extend neurites to establish intricate communication networks, with synapses serving as the core structures that mediate intercellular signal transmission. Given that the molecular mechanisms underlying electrical synapse formation are relatively less understood than those of chemical synapses, contemporary research focus remains heavily directed toward the structural composition and organizing pathways of chemical synapses (Yogev and Shen, 2014; Portman et al., 2026).

**Figure 1:**
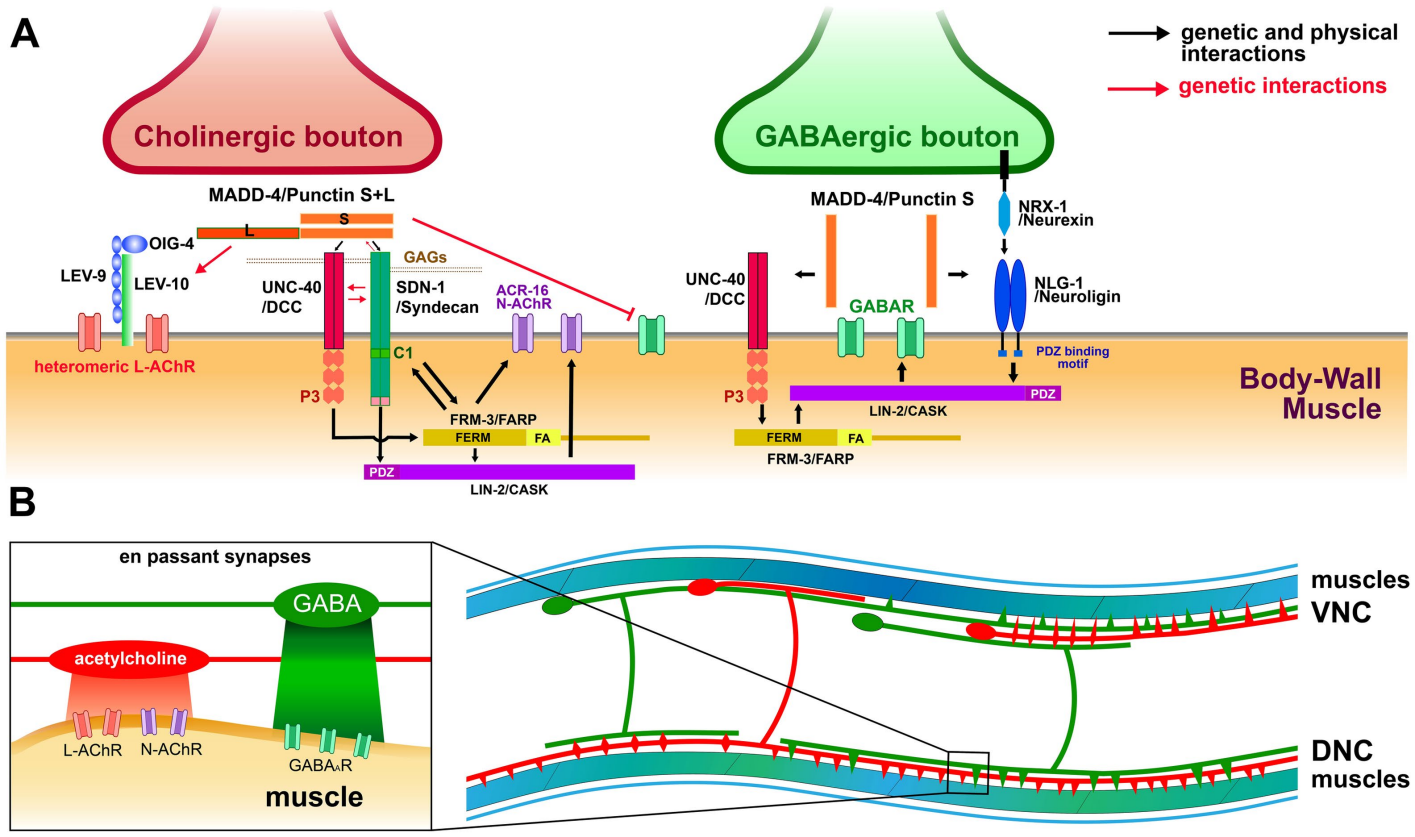
The neuromuscular system of *C. elegans*. **(A)** Assembly of local postsynaptic protein complexes. **(B)** Schematic topology of the *C. elegans* neuromuscular network. VNC, ventral nerve cord; DNC, dorsal nerve cord; GABA, gamma-aminobutyric acid; S/L, short/long (isoforms); DCC, deleted in colorectal cancer; FERM, protein4.1, Ezrin, Radixin, Moesin; FA, FERM-Adjacent; PDZ, PSD-95/Dlg/ZO-1; GAGs, glycosaminoglycans; LIN-2, lineage-2; CASK, calmodulin-dependent serine protein kinase.

The mononucleated body wall muscle cells in *C. elegans* are innervated by motor neuron processes extending along the dorsal and ventral nerve cords. These neurons largely exhibit an unmyelinated, unipolar morphology, forming synapses *en passant* along adjacent parallel neurites (Witvliet et al., 2021). High-resolution electron microscopy reveals the intricate synaptic microstructures of these networks, characterized by distinct presynaptic dense projections, surrounding clear synaptic vesicles containing neurotransmitters, and dense-core vesicles that control neuropeptide release (Mizumoto et al., 2023). To establish these networks, *C. elegans* body wall muscles extend “muscle arms”—cytoplasmic processes analogous to neuronal dendrites—through which each cell receives dual inputs from excitatory cholinergic and inhibitory gamma-aminobutyric acid-ergic (GABAergic) motor neurons. This unique neuromuscular junction architecture offers an ideal model for investigating how distinct neurotransmitter receptors are sub-compartmentalized and concentrated at corresponding release sites (Calahorro and Izquierdo, 2018). Within this structural framework, muscle arm development defective-4 (MADD-4) functions as a crucial anterograde synaptic organizing factor that ensures precise structural and functional alignment between presynaptic neurotransmitter release zones and postsynaptic receptors. Consequently, a single deletion of MADD-4 significantly reduces the clustering of synaptic neuroligin-1 (NLG-1) and Type-A GABA receptors (GABA_A_ Rs), whereas a double deletion of MADD-4 and Neurexin-1 causes complete receptor dispersal, thereby disrupting inhibitory synaptic transmission (Maro et al., 2015). The *C. elegans* synaptic signaling network achieves this precise regulation through the differential actions of distinct MADD-4 isoforms. At excitatory cholinergic synapses, motor neurons secrete the long isoform, MADD-4 L, to induce the clustering of two distinct receptor types. Levamisole-sensitive acetylcholine receptors (L-AChRs) rely on the assistance of an extracellular protein complex including LEVamisole-resistant 9 (LEV-9), LEVamisole-resistant 10 (LEV-10), and One ImmunoGlobulin domain protein 4 (OIG-4). In contrast, nicotine-sensitive acetylcholine receptors (N-AChRs) depend on the transmembrane proteins syndecan-1 (SDN-1) and uncoordinated-40 (UNC-40) for their proper synaptic localization (Hendi et al., 2019). Conversely, at inhibitory GABAergic synapses, the short isoform MADD-4S drives the postsynaptic clustering of GABA_A_ Rs through two parallel pathways: it directly binds the cell adhesion molecule NLG-1 and recruits UNC-40 to support receptor localization (Tu et al., 2015). This process, whereby UNC-40 assembles the intracellular postsynaptic scaffold, is strictly orchestrated by MADD-4S (Maro et al., 2015; Xu et al., 2015; Zhou et al., 2020). This sophisticated dual-isoform mechanism elegantly demonstrates how mononucleated muscle cells utilize partially overlapping core components to specifically segregate and assemble postsynaptic microdomains corresponding to different neurotransmitters on the same plasma membrane (Jin, 2015; Bhandari et al., 2024). The molecular organization of *C. elegans* neuromuscular junctions and postsynaptic domains is illustrated in Figure 1.

### 2.2 Neurotransmitter and neuropeptide signaling networks

Neurons release classical neurotransmitters in tandem with multiple neuropeptides, which integrate within neural circuits to cooperatively orchestrate behavioral outputs. Accumulating evidence demonstrates that small-molecule and neuropeptide cotransmission pairs are conserved across both invertebrate and vertebrate lineages. Consequently, *C. elegans* serves as a premier genetic model for delineating the architecture of neuropeptide signaling networks and the mechanistic underpinnings of behavioral regulation (Nusbaum et al., 2017). Table 1 summarizes the primary neurotransmitters and neuromodulators in *C. elegans*.

**Table 1:** Major neurotransmitters and neuromodulators in *C. elegans*.

Category	Neurotransmitter/neuropeptide	Key markers (Genes)	Primary functions	References
Classical neurotransmitters	Acetylcholine	ChAT (*cha-1*) VAChT (*unc-17*)	Primary excitatory neurotransmitter; drives body wall muscle contraction; maintains pharyngeal pumping rhythm; regulates egg-laying and mating behaviors.	Cook et al. (2020), Duerr et al. (2021), Emerson et al. (2021)
	GABA	GAD (*unc-25*) VGAT (*unc-47*)	Primary inhibitory neurotransmitter; maintains coordinated body bending; triggers defecation; restricts head foraging amplitude.	Yemini et al. (2021), Wang et al. (2024), Yue et al. (2024)
	Glutamate	VGLUT (*eat-4*)	Sensory perception; associative learning and memory; formulation of foraging strategies.	Sato et al. (2021), Taylor et al. (2021)
Biogenic amines	Dopamine	TH (*cat-2*)	Mechanosensation; habituation learning.	Kindt et al. (2007), Han et al. (2017), Millet et al. (2025)
	Serotonin	TPH (*tph-1*)	Enhances pharyngeal pumping; mediates food-induced slowing response; promotes egg-laying.	Flavell et al. (2013), Dag et al. (2023)
	Tyramine	TDC (*tdc-1*)	Promotes escape responses; inhibits exploratory head swinging.	Donnelly et al. (2013)
	Octopamine	TBH (*tbh-1*)	Inhibits egg-laying during starvation; antagonizes 5-HT signaling; promotes food-seeking behavior.	Fernandez et al. (2020)
Neuropeptides	FMRFamide-like peptides	*flp* family (*flp-1* ~ *flp-34*)	Induces systemic quiescence for physiological protection under stress; fine-tunes locomotion rate, turning frequency, and egg-laying.	Nelson et al. (2014), Ripoll-Sánchez et al. (2023), Watteyne et al. (2024)
	Neuropeptide-like proteins	*nlp* family (*nlp-1* ~ *nlp-82*)	Balances sensory perception and foraging behaviors; regulates homeostasis during stress responses.	Fadda et al. (2020), Peng et al. (2023), Ripoll-Sánchez et al. (2023)
	Insulin-like peptides	*ins* family (*ins-1* ~ *ins-39*, *daf-28*)	Regulates development, metabolism, lifespan, learning, and memory.	Cho et al. (2016), Zheng et al. (2018), Tomioka et al. (2022)

ChAT, choline acetyltransferase; GABA, γ-aminobutyric acid; NLPs, neuropeptide-like proteins; FLPs, FMRFamide-like peptides; ILPs, insulin-like peptides; VAChT, vesicular acetylcholine transporter; GAD, glutamate decarboxylase; VGAT, vesicular inhibitory amino acid transporter; VGLUT, vesicular glutamate transporter; TH, tyrosine hydroxylase; TPH, tryptophan hydroxylase; TDC, tyrosine decarboxylase; TBH, tyramine β-hydroxylase; PC2, proprotein convertase 2; CPE, carboxypeptidase E.

#### 2.2.1 Classical neurotransmitters

Acetylcholine, glutamate, and GABA collectively form the foundation of rapid signal transduction within nematode neural circuits. Acetylcholine acts on ligand-gated ion channels to mediate muscle contraction and regulate various cognitive and addictive behaviors. The *C. elegans* genome encodes 29 acetylcholine receptor subunits. However, the assembly patterns and specific physiological mechanisms of most neuronal receptors remain incompletely elucidated and warrant further investigation (Zhao J. et al., 2026). GABA is classically recognized as a primary inhibitory signal. Recent findings reveal an unexpected paradigm in *C. elegans*. GABA released by D-type motor neurons exerts an excitatory—rather than inhibitory—effect on A-type motor neurons to coordinate backward locomotion. Yet, the precise organizational and synaptic scaffolding mechanisms of these atypical excitatory GABA receptors within the motor circuit remain poorly defined (Wang et al., 2026). Glutamate serves as the principal excitatory neurotransmitter. To investigate its behavioral consequences while bypassing direct, acute neurotoxic cell death, researchers have utilized *C. elegans* to construct a glutamate spillover model. By inactivating the glial glutamate transporter GLT-1, they precisely delineated the specific impacts of synaptic glutamate spillover on nematode behavior (Katz et al., 2019). However, whether these chronic behavioral shifts in transport-deficient nematodes accurately model the progressive, non-cell-autonomous excitotoxicity seen in mammalian neurodegeneration remains a critical challenge.

#### 2.2.2 Biogenic amine neurotransmitters

High-performance liquid chromatography enables the isolation and identification of four primary biogenic amine neurotransmitters in *C. elegans*: octopamine, dopamine, serotonin, and tyramine. Collectively, these transmitters enable the nematode to modulate behavioral motivation in response to environmental changes (Chase and Koelle, 2007). Octopamine acts as an analog of vertebrate norepinephrine. By dissecting the neural circuits downstream of octopaminergic neurons in invertebrates such as *C. elegans*, researchers found that starvation upregulates octopamine synthesis (Tao et al., 2016). This elevation subsequently mediates diverse physiological and behavioral processes, including lipid metabolism, stress responses, egg-laying, and foraging (Zhao H. et al., 2026). Synthesized via the catalytic action of tyrosine hydroxylase, dopamine regulates complex behaviors, including locomotion, learning, and habituation to environmental signals. Because the dopaminergic network is highly conserved, *C. elegans* serves as a critical model organism to decipher the molecular mechanisms of aging and to simulate neurodegenerative conditions such as Parkinson’s disease (Muralidhara and Hardege, 2025). The serotonin system primarily originates from three specific neuron pairs: neurosecretory motor neuron (NSM), amphid sensory neuron (ADF), and hermaphrodite specific neuron (HSN). NSM detects food availability and releases serotonin to stimulate feeding, motor slowing, and dwelling behavior. ADF senses bacterial metabolites to modulate acute feeding and learned avoidance responses against pathogenic bacteria. Located in the midbody, HSN neurons orchestrate egg-laying and locomotion rhythms (Feng et al., 2025). As another vital invertebrate trace amine closely biosynthetically linked to octopamine, tyramine is synthesized via the catalytic action of tyrosine decarboxylase. Upon external stimulation, the TA signaling system rapidly responds to diverse stressors, playing a central role in mediating fight-or-flight-like stress behaviors (Rosikon et al., 2023).

#### 2.2.3 Neuropeptide

The diverse and functionally complex neuropeptides in *C. elegans* constitute an extensive extrasynaptic signaling network that is structurally distinct from synaptic and monoaminergic systems. These neuropeptides are primarily classified into three major categories: FLPs, NLPs, and ILPs (Beets et al., 2023). Specifically, FLPs function as central regulators of neuromuscular activity; although they typically suppress nervous system activity in most behavioral assays, pharmacological evidence indicates they can also exert excitatory effects (Li and Kim, 2014). Encompassing over 40 distinct genes that encode more than 80 mature peptides, the *nlp* gene family exhibits profound functional diversity and is strongly hypothesized to modulate feeding behavior due to its prominent expression in *C. elegans* pharyngeal neurons (Papaioannou et al., 2008). Insulin-like peptides directly regulate animal metabolism, growth, and development, and *C. elegans* research has been essential for deciphering these conserved molecular mechanisms. The nematode possesses a single canonical insulin/IGF-1 receptor, DAF-2. Current models suggest that the expansive repertoire of insulin-like (INS) peptides transduces signals predominantly through this receptor (Zhu and Chin-Sang, 2024). While the comprehensive resources accumulated for *C. elegans*—spanning synaptic connectivity, whole-brain neural activity, and molecular expression profiles—provide robust data support for network modeling, translating these multi-omic datasets into predictive functional maps remains a major challenge.

## 3 Modeling neurodegenerative diseases in *C. elegans*

Neurodegenerative diseases constitute a major public health challenge affecting a substantial portion of the global population. Alzheimer’s disease (AD), Parkinson’s disease (PD), amyotrophic lateral sclerosis (ALS), and Huntington’s disease (HD) represent the most prevalent forms worldwide. *C. elegans* serves as an essential bridge between *in vitro* assays and mammalian models. Leveraging its distinct capacity for high-throughput *in vivo* screening, this nematode significantly accelerates the candidate drug development pipeline (Panda et al., 2025). To provide a comprehensive overview, Table 2 summarizes the established *C. elegans* models utilized for these neurodegenerative conditions.

**Table 2:** Overview of *C. elegans* models for neurodegenerative disorders.

Disease	Strain (Promoter::Transgene)	Transgene expression	Phenotype	Limitations	Reference
Alzheimer’s disease	CL2006 *(unc-54p::human Aβ_1-42_)*	Constitutive expression in muscle cells	Progressive, adult-onset paralysis. Intramuscular Aβ deposits, shortened lifespan.	Limited to muscle toxicity, cannot mimic central cell-to-cell propagation.	Link (1995), Alvarez et al. (2022)
	GRU102 *(myo-2p::YFP+unc-119p::Aβ_1-42_)*	Constitutive pan-neuronal expression	Impaired neuromuscular, sensorimotor behavior.	Lack of neuron-type specificity, low-throughput behavioral scoring.	Fong et al. (2016), Tiwari et al. (2024)
	CL4176 *(myo-3p::human Aβ_1-42_)*	Constitutive expression in muscle cells	Temperature-induced paralysis and progeny arrest; Roller phenotype.	Temperature-sensitive, low batch reproducibility.	Drake et al. (2003), Caldero-Escudero et al. (2024)
	CL2122 *(unc-54p::SP::human Aβ_1-42_)*	Constitutive expression in muscle cells	Slow adult movement. Intramuscular Aβ deposits.	Muscle-specific expression, lacks neuronal microenvironment.	Fay et al. (1998), Navarro-Hortal et al. (2022)
	CL2331 *[myo-3p::GFP::A-Beta (3–42)]*	Constitutive expression in muscle cells	Low brood size. Sicker at higher temperatures	Non-specific toxicity phenotypes limit its utility for high-throughput drug screening.	Link et al. (2008), Wen et al. (2024)
	CL2355 *(snb-1p::SP::human Aβ_1-42_::long 3′UTR)*	Inducible pan-neuronal expression	Memory deficits, abnormal thrashing in liquid, partial sterility.	Non-specific pan-neuronal vulnerability, low-throughput, highly variable cognitive assays	Luo et al. (2009), Sillapakong et al. (2025)
	GMC101 *(unc-54p::Aβ_1-42_)*	Constitutive expression in muscle cells	Temp-induced paralysis & muscular Aβ_1-42_ aggregation.	Muscle expression lacks neuronal context, temp-induced thermal stress.	McColl et al. (2012), Lin et al. (2022), Liu L. et al. (2025)
	CL2120 *(unc-54p::human Aβ_1-42_)*	Constitutive expression in muscle cells	Aβ expression and fibrillation; temperature-enhanced toxicity.	Developmental confounding from constitutive expression.	Fay et al. (1998), Di Carlo (2012)
	CK10 *(aex-3p::h4R1NTauV337M; myo-2p::gfp)*	Constitutive pan-neuronal expression	Progressive uncoordinated movement, insoluble phosphorylated Tau aggregates.	Lacks circuit specificity, constitutive expression risks developmental confounding.	Kraemer et al. (2003), Kow et al. (2023)
	PIR5 *pirIs5[snb-1p::htau40A152T-low; myo-2p::gfp]*	Constitutive pan-neuronal expression	Synaptic transmission impairments, reduced lifespan; independent of protein aggregation.	Lacks circuit specificity, constitutive expression risks developmental confounding.	Pir et al. (2016)
	CK2620 *(snb-1p::hTMEM106b-core+ myo-3p::mCherry)*	Constitutive pan-neuronal expression	Severe progressive locomotor deficits, extensive cytoplasmic protein aggregation.	Lacks full-length transmembrane architecture, alters native proteotoxicity profiles.	Riordan et al. (2025)
Parkinson’s disease	NL5901 *(unc-54p::alpha-synnuclein::YFP)*	Constitutive expression in muscle cells	Muscular expression of α-synuclein-YFP and *in vivo* aggregation.	No dopaminergic neuronal microenvironment	van Ham et al. (2008), Chen M. et al. (2024)
	BY273 *(dat-1p::alpha-synuclein)*	Dopaminergic neurons	Progressive dopaminergic neuronal loss with neurite blebbing	Promoter downregulation risks false-positive cell loss scoring	SenGupta et al. (2021), Tiwari et al. (2024)
	UA44 *(dat-1p::alpha -syn high)*	Specific expression in dopaminergic neurons	Locomotion reduced, neuron degeneration	Localized to DA neurons, missing pan-neuronal networks; prone to promoter-silencing artifacts	Gaeta et al. (2023), Griffin and Owens (2025)
	DDP1 *(unc-54p::alpha -syn::CFP)*	Constitutive expression in muscle cells	Reduced lifespan and pharyngeal pumping; *in vivo* α-synuclein aggregation.	Lacks neuronal specificity	Bodhicharla et al. (2012), Maulik et al. (2017)
	OW13 *(unc-54p::alpha -syn::YFP)*	Constitutive expression in muscle cells	Muscular α-syn aggregation	Muscle-specific expression lacks neuronal microenvironment	van Ham et al. (2008), Brunetti et al. (2020)
	BZ555 *(dat-1p::GFP)*	Specific expression in dopaminergic neurons	GFP-labeled dopaminergic neurons; 6-OHDA resistant.	Signals protein down-regulation/synaptic failure, not true cell death.	Nass et al. (2002), Limke et al. (2023)
Amyotrophic lateral sclerosis	CK422 *[snb-1p::TDP-43(G290A), myo-2p::dsRED]*	Pan-neuronal expression	Pan-neuronal TDP-43 aggregation, severe motor dysfunction, shortened lifespan.	Lacks motor neuron specificity; rapid decline narrows experimental window.	Liachko et al. (2010), Garcia-Toscano et al. (2024)
	BR5270 *(rab-3p::F3ΔK280 + myo-2p::mCherry)*	Constitutive pan-neuronal	Neuronal Tau aggregation, impaired chemotaxis and locomotion.	Lacks cell-type specificity; fragment expression misses full-length tau interactions.	Fatouros et al. (2012), Chen L. et al. (2024)
	XQ207 *xqIs133 [unc- 47p::TDP-43(A315T); unc-119(+)]*	Constitutive expression in GABAergic motor neurons	Insoluble TDP-43/FUS aggregation and neuronal degeneration, adult-onset progressive paralysis, normal lifespan.	Lacks cholinergic motor neuron targeting; manual touch-response assays impede high-throughput screening.	Vaccaro et al. (2012), Pir et al. (2026)
	AM263 *[unc-54p::Hsa-sod-1 (WT)::YFP]*	Constitutive expression in muscle cells	WT SOD-1 expression, mild age-dependent aggregation.	Non-neuronal targeting; lacks robust baseline phenotypes; fusion tag artifacts.	Gidalevitz et al. (2009), Liu et al. (2018)
	PJH897 *[rgef-1p::FUS (P525L)]*	Constitutive pan-neuronal	Cytoplasmic FUS aggregation, shortened lifespan, severe paralysis.	Pan-neuronal non-specificity; narrow experimental window; missing glial cross-talk.	Murakami et al. (2012)
	*HA2987* *sod-1* *(* *rt449* *[G93AC]) II*	Endogenous expression	Axonal degeneration in GABAergic motor neurons, progressive paralysis.	Mild baseline neurodegeneration; ubiquitous expression lacks cell-type specificity.	Baskoylu et al. (2022), Tossing et al. (2022)
	ZM5844 *(rgef-1p::FUSP525L::GFP)*	Pan-neuronal expression	Mutant FUS expression in neurons, reduced motor activity.	Lacks motor neuron specificity	Murakami et al. (2012), Alirzayeva et al. (2024)
	GA801 *wuIs152 contains [sod-1(genomic)]*	Constitutive ubiquitous expression	Roller phenotype; increased protein oxidative damage and altered oxidative stress resistance	No baseline pathology; rol-6 marker confounds motility assays; lacks tissue-specific resolution.	Doonan et al. (2008), Cabreiro et al. (2011)
Huntington’s disease	AM138 *(unc-54p::Q24::YFP)*	Constitutive expression in muscle cells	Diffuse distribution of soluble Q24-YFP without toxicity.	Lacks protein aggregation pathology; body-wall muscle expression limited.	Morley et al. (2002)
	HA759 *(osm-10p::HtnQ150)*	Specific expression in ASH sensory neurons	Accelerated ASH neuronal death, larval onset.	Limited ASH neuron specificity; rol-6 background disrupts normal locomotion.	Bicca Obetine Bicca Obetine Baptista et al. (2020)
	AM141 *(unc-54p::Q40::YFP)*	Constitutive expression in muscle cells	Age-dependent Q40-YFP aggregation	Body-wall muscle expression limited.	Morley et al. (2002), Vela et al. (2022)
	AM101 *(F25B3.3p::Q40::YFP)*	Pan-neuronal expression	Neuron degeneration, mild motor defects.	Absence of robust behavioral phenotypes; nerve ring imaging resolution limited.	Brignull et al. (2006), Redweik and Xue (2025)
	AM716 *(F25B3.3p::Q67::YFP)*	Pan-neuronal expression	Neuronal polyQ67 aggregation, age-dependent loss of motility.	Densely packed neural imaging limitations; high genetic background vulnerability to silencing.	Brignull et al. (2006), Lee et al. (2023)
	AM140 *(unc-54p::Q35::YFP)*	Constitutive expression in muscle cells	Muscle polyQ35 aggregation, age-dependent motor dysfunction.	Borderline threshold causing high phenotypic variability; body-wall muscle expression limited.	Morley et al. (2002), Wu Y. et al. (2025)
	EAK103 *[unc-54p::Htt513(Q128)]*	Constitutive expression in muscle cells	Muscle polyQ128 aggregation, motility defect.	Limited to body-wall muscle expression; background confounding pure polyQ pathways.	Lee et al. (2017), Liu et al. (2024)

Notes: This table summarizes the *C. elegans* models commonly used in the study of AD, PD, ALS, and HD. Aβ_1-42_, Amyloid beta1-42; *α*-syn, Alpha-synuclein; Htt / Htn, Huntingtin; Hsa, *Homo sapiens* (Human); TDP-43, TAR DNA-binding protein 43; FUS, Fused in sarcoma; Temp, Temperature; YFP, Yellow Fluorescent Protein; GFP, Green Fluorescent Protein; SOD-1, Superoxide dismutase 1; CFP, Cyan Fluorescent Protein; SP, Signal Peptide; WT, Wild Type; dsRED, *Discosoma* sp. red fluorescent protein; 6-OHDA, 6-Hydroxydopamine; P indicates promoter (e.g., *P*unc-54 represents the *unc-54* promoter); polyQ/Q, Polyglutamine/Glutamine (e.g., Q40 represents a tract of 40 glutamines); UTR, Untranslated region (e.g., 3′UTR).

### 3.1 Alzheimer’s disease

Alzheimer’s disease represents a chronic, progressive neurological disorder that currently affects over 57 million people worldwide, accounting for 60 to 70% of all dementia cases and generating nearly 10 million novel incident cases annually (World Health Organization, 2025, 3.31). As the disease advances, pathological changes sweep through an array of brain areas, heavily impacting the entorhinal cortex and hippocampus before extending to the prefrontal cortex and the broader limbic network (Anand et al., 2025). The primary pathological signatures of the disease are characterized by the extracellular aggregation of amyloid-beta plaques, alongside the formation of neurofibrillary tangles within neurons (Zheng and Wang, 2025). Neurofibrillary tangles (NFTs) consist primarily of hyperphosphorylated tau. Physiologically, tau promotes tubulin assembly into microtubules and stabilizes these structural filaments (Wang and Mandelkow, 2016). Extracellular plaques comprise aggregated Aβ peptides. Following extracellular accumulation, neurotoxic Aβ oligomers undergo endocytotic uptake. This internalization induces tau phosphorylation and subsequent NFT formation (Alvarez et al., 2022). Mechanistically, aberrant Aβ accumulation likely triggers a pathogenic cascade. It drives abnormal tau phosphorylation and NFT assembly, destabilizing microtubules and obstructing axonal transport to precipitate neuronal dysfunction and death (Hardy and Selkoe, 2002; Mandelkow and Mandelkow, 2012). Both Aβ plaques and NFTs independently provoke reactive oxygen species generation, exacerbating neurotoxic damage (Tiwari et al., 2019; Wu et al., 2023). *C. elegans* lacks endogenous orthologs for the amyloid precursor protein and its cleavage enzyme, β-secretase. This means that *C. elegans* cannot produce Aβ naturally. Consequently, the AD model in *C. elegans* is mainly set up by driving transgenic expression of Amyloid-β transgenes, tau proteins, or both of them (Fang et al., 2019). The precise molecular pathology of AD remains incompletely understood despite decades of research. Accelerated by the aging global population, this disorder has evolved into a severe public health challenge (da Silva et al., 2026).

#### 3.1.1 Amyloid-β models

The initial *C. elegans* AD model utilized the unc-54 promoter to drive the expression of Aβ peptides containing a secretory signal in body wall muscles, intending to simulate extracellular amyloid deposition. This transgenic nematode exhibited a characteristic paralysis phenotype. It established a highly efficient quantitative model for evaluating Aβ toxicity and screening potential therapeutics (Chen and Zhang, 2022). The endogenous *C. elegans* amyloid precursor protein homolog (APL-1) lacks the human Aβ sequence. Because the APL-1 protein differs in amino acid sequence from the beta-amyloid peptide region in human APP, it cannot generate beta-amyloid peptides. This trait ensures a clean endogenous background, rendering the nematode an ideal *in vivo* model to investigate the specific toxicity of human A*β* (Daigle and Li, 1993). Early modeling efforts, hindered by aberrant cleavage of synthetic signal peptides, yielded truncated Aβ_3-42_.

Current transgenic *C. elegans* strains are primarily constructed to specifically express Aβ_1-42_ in body wall muscle cells, as this fragment is considered the most toxic Aβ isoform (McColl et al., 2009; Roussos et al., 2023). Inserting an Asp-Ala (DA) sequence at the N-terminus of the human Aβ sequence achieves full-length Aβ_1-42_ expression in nematode muscles. This drives the accumulation of soluble oligomers and induces severe progressive paralysis, mirroring classic degenerative phenotypes (McColl et al., 2012). To dissect oligomer formation and its pathogenic consequences, an optogenetic model was developed. This system expresses a fluorescently tagged Aβ protein in vivo that rapidly oligomerizes upon blue light illumination (Timofeeva et al., 2025). RNAi screening in muscle-expression models demonstrated that inhibiting mitochondrial ferritin-1 attenuates mitochondrial reactive oxygen species (ROS) levels. This intervention effectively reduces paralysis rates and extends nematode lifespan (Huang et al., 2018). However, such technological intricacy does not obscure the physiological simplicity of *C. elegans*, which carries inherent translational drawbacks. AD is a human neurological disorder, yet the most commonly used high-throughput drug screening models in *C. elegans* express Aβ within the muscles. The mechanisms of drugs screened in the muscles may fundamentally fail to explain synaptic regression or neuronal death. Furthermore, *C. elegans* lacks endogenous β-secretase and therefore cannot fully simulate the complex proteolytic processing dynamics of amyloid precursor protein inside the human body.

#### 3.1.2 Microtubule-associated protein tau (MAPT)models

The intracellular accumulation of aberrantly phosphorylated Tau and the subsequent formation of NFTs represent a core pathological hallmark of Alzheimer’s disease and other age-related neurodegenerative disorders (Scheltens et al., 2021). Tau is a microtubule-associated protein. It primarily functions to stabilize the microtubule network. In humans, this protein is encoded by the single-copy MAPT gene, which comprises 16 exons and is located on chromosome 17q21 (Pir et al., 2017). As the sole identified Tau homolog in *C. elegans*, PTL-1 shares high structural conservation with human Tau. This protein is indispensable for maintaining nervous system homeostasis throughout the nematode lifespan and regulating age-associated neurodegeneration (McDermott et al., 1996). Loss of ptl-1 function reduces the number of viable offspring and impairs touch sensitivity; however, normal development remains unaffected. By generating transgenic nematode models expressing human Tau, researchers can accurately recapitulate Tau hyperphosphorylation and conformational pathology *in vivo* (Pir et al., 2017; Natale et al., 2020). These models are highly effective for identifying genetic modifiers of AD and tauopathies and elucidating the intercellular transmission mechanisms of pathogenic proteins. They also provide a robust screening platform to rapidly isolate drug candidates capable of lowering pathological Tau levels (Sandhof et al., 2025; Carroll et al., 2026). Nevertheless, these systems carry distinct limitations regarding physiological fidelity. Most transgenic strains utilize pan-neuronal promoters to drive human Tau overexpression, which creates non-physiological toxicity artifacts and precipitates acute cellular collapse.

### 3.2 Parkinson’s disease

Parkinson’s disease ranks as the second most prevalent neurodegenerative disorder worldwide. The disease exhibits a general population prevalence of approximately 0.3%, which escalates to over 3% in individuals aged 80 and older. Excluding rare early-onset cases, it predominantly afflicts the elderly demographic (Ye et al., 2023). Progressive loss of dopaminergic neurons and subsequent motor dysfunction constitute its core clinical features. Clinicians currently face a dual challenge: the absence of reliable biomarkers for early diagnosis and the lack of disease-modifying therapies capable of reversing the disease course. At the cytopathological level, misfolded fibrillar *α*-synuclein (α-syn) pathologically aggregates within neurons to form Lewy bodies. This intracellular accumulation represents the classic hallmark driving the neurodegenerative process (Redl et al., 2025). A complex pathogenic cascade governs PD onset and progression at deeper molecular dimensions. Oxidative damage triggered by mitochondrial complex I defects, impaired protein degradation pathways, and sustained chronic neuroinflammation intertwine to construct the core pathogenic network of the disease (Chaudhary et al., 2025). α-Synuclein regulates synaptic vesicle assembly, and its encoding gene, *SNCA* (*PARK1*/*PARK4*), was the first identified causative gene for familial PD. Genetic mutations, duplications, or triplications of *SNCA* directly manifest as autosomal dominant Parkinson’s disease (Cooper and Van Raamsdonk, 2018).

*C. elegans* retains an evolutionarily conserved neurotransmission system. The nematode synthesizes dopamine through exactly eight dopaminergic neurons. It provides a robust platform for evaluating neurotoxin effects and offers established transgenic strains expressing human α-synuclein. Orthologs of human PD-associated genes—including lrk-1, pink-1, pdr-1, djr-1.1, and catp-6—are present and functionally intact within this model (da Silva et al., 2024). To quantify nematode behavioral phenotypes and screen for therapeutics capable of alleviating PD-like motor dysfunction, researchers developed the machine-learning algorithm CeSnAP (*C. elegans* Snapshot Analysis Platform). Utilizing this platform, a high-throughput thrashing analysis was conducted on over 17,000 nematodes to evaluate a library of 50 FDA-approved drugs. This large-scale screen successfully identified enasidenib, ethosuximide, metformin, and nitisinone as highly promising candidate therapeutics for late-stage PD intervention (Sohrabi et al., 2021). Recent studies reveal that metformin suppresses the TLR4/MyD88/NF-κB pathway, coupling neuroinflammation attenuation with copper homeostasis regulation—a mechanism distinct from ferroptosis. Metformin reduces intracellular copper accumulation and downregulates cuproptosis-associated proteins (FDX1 and SLC31A1). This alleviates copper-dependent proteotoxic stress, thereby blocking cuproptosis and protecting dopaminergic neurons in PD models (Shang et al., 2025).

However, *C. elegans* lacks the complex nigrostriatal pathway characteristic of the human brain. Motor impairments such as a reduction in curling or thrashing frequencies can hardly be mapped fully onto human clinical symptoms like resting tremors or bradykinesia. Furthermore, these models present distinct spatiotemporal limitations in simulating disease progression. While models induced by neurotoxins such as 6-OHDA represent acute neurotoxic phenotypes, authentic PD is a progressive, degenerative process spanning decades.

### 3.3 Amyotrophic lateral sclerosis

Amyotrophic lateral sclerosis (ALS), also known as Charcot’s disease or Lou Gehrig’s disease, is a devastating motor neuron disease (MND). Its core pathological feature involves the progressive degeneration and retraction of motor nerve terminals from target muscles, subsequently affecting upper and lower motor neurons that control voluntary musculature within the central nervous system (Kwaśniewska et al., 2026). Clinically, patients present with muscle stiffness and progressive weakness of the limbs and bulbar muscles, inexorably leading to varying degrees of speech, swallowing, and respiratory dysfunction (Katz et al., 2026). Mechanistically, the vast majority of ALS cases are sporadic; only about 10% are familial (fALS). Genomic variations in the superoxide dismutase 1 (*SOD1*) gene constitute the primary causative factor for fALS, accounting for approximately 20% of these familial cases (O'Neill et al., 2025; Xie et al., 2025). Mutations in the fused in sarcoma (*FUS*) gene—which encodes an essential DNA- and RNA-binding protein critical for transcription, splicing, transport, and genomic stability—account for approximately 4% of familial instances (Vance et al., 2009; Dormann et al., 2010). Approximately 3% of familial ALS cohorts are characterized by aberrations in TDP-43. This ubiquitous 43 kDa nucleic acid-binding protein serves as a master regulator of transcriptional control, alternative mRNA splicing, and RNA stability (Lagier-Tourenne and Cleveland, 2009).

To comprehensively dissect the intricate pathogenic mechanisms of ALS, researchers have widely utilized transgenic *C. elegans* models, which are systematically summarized in Table 2. Featuring highly conserved cellular stress and pro-survival pathways, alongside unique advantages for delineating neural activities—such as processing sensory information, integrating motor circuits, and modulating behavioral states—this nematode serves as an exceptional organismal platform for modeling ALS (Prova et al., 2026). Utilizing these models, numerous studies have confirmed that widespread dysregulation across multiple signaling pathways drives disease progression. This encompasses loss-of-function mutations in mitochondria-associated genes, viral-mediated microRNA dysregulation, and pathological cascades involving the inflammatory mediator NF-κB (Källstig et al., 2021; Meijboom and Brown, 2022; Menge et al., 2025). Driven by these progressive mechanistic insights, leveraging *C. elegans* models to investigate deficits in energy metabolism has burgeoned into a pivotal front-line research direction, which will significantly facilitate the development of novel therapeutic strategies for ALS (Tefera et al., 2021). Nevertheless, *C. elegans* lacks a complex circulatory system, distinct vascular structures, and mammalian-like organ-to-organ metabolic crosstalk. This inherently limits its capability to fully recapitulate systemic energy redistribution and the multi-tissue metabolic collapse observed in higher mammalian models.

### 3.4 Huntington’s disease

**Figure 2:**
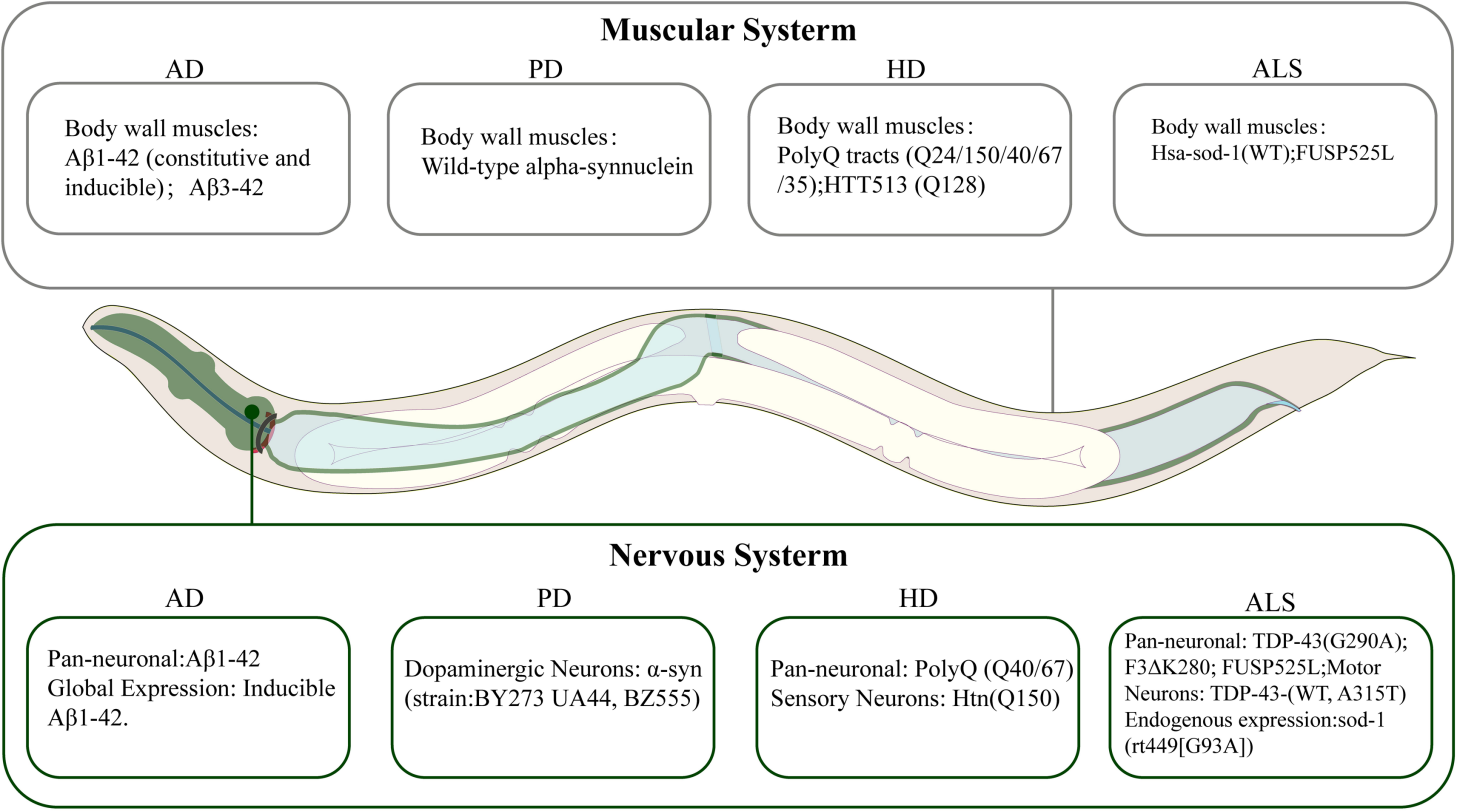
Simplified anatomical schematic of *C. elegans*. Schematic diagram of *C. elegans* anatomy illustrating the specific tissue distribution of transgenic expression in the discussed disease models. The targeted sites are categorized into two primary physiological systems, which are further classified by four distinct neurodegenerative disorders. The nervous system is highlighted in green, whereas the muscular system is depicted in gray.

Huntington’s disease is a progressive neurodegenerative disorder with autosomal dominant inheritance. At the molecular level, pathogenesis is driven by an abnormal CAG trinucleotide repeat expansion (typically ≥35), which generates a cytotoxic polyglutamine (polyQ) tract at the N-terminus of the huntingtin (HTT) protein. The length of this polyQ tract correlates strictly with both the age of onset and overall disease severity. Pathological aggregates accumulate continuously within neuronal somata over decades; because frank neurodegeneration erupts only after crossing a critical pathogenic threshold, patients experience a distinctly prolonged preclinical phase (El Din and Thabit, 2024; Scahill et al., 2025). During this protracted disease course, non-motor symptoms such as insomnia, sleep fragmentation, and circadian rhythm disruptions are highly prevalent. Clinical studies confirm that patients frequently exhibit significantly reduced nocturnal melatonin levels alongside delayed secretion peaks (Gu et al., 2026). To model these pathological features, transgenic *C. elegans* strains have been widely utilized, wherein polyQ tracts are typically expressed specifically within sensory neurons. These nematode models successfully recapitulate core disease features, including progressive protein aggregation and neurodegeneration. Through these *in vivo* systems, researchers have identified multiple conserved neuroprotective mechanisms; these defense systems primarily involve proteostasis networks, encompassing molecular chaperones, autophagic components, and the insulin/IGF-1 signaling pathway (Xu et al., 2025). However, although these nematode models provide essential insights for identifying novel drug targets, they possess a critical limitation: *C. elegans* lacks an endogenous *HTT* ortholog. Furthermore, pronounced mechanistic discrepancies exist across diverse animal species. Future efforts must prioritize the generation of animal models expressing full-length mutant HTT and mandate rigorous cross-species validation, which is imperative to bridge the translational gap between basic discovery and clinical application (Sandhof et al., 2020). Methodologically, while restricting polyQ expression to localized tissues facilitates high-throughput scoring, it might obscure complex, network-wide pathogenic cascades. Furthermore, relying on isolated short fragments rather than the full-length HTT macromolecule could potentially simplify aggregation kinetics and alter cytotoxicity profiles. Consequently, phenotypic rescues in these simplified platforms may not fully mirror therapeutic efficacy in higher organisms, highlighting the need for cautious extrapolation of drug-screening data. The schematic illustration of the transgenic expression strategies across these distinct disease models is presented in Figure 2.

## 4 Translational applications and frontier technology prospects of *C. elegans*

### 4.1 Optimization of screening models

Historically, traditional drug screening models have long struggled to reconcile high throughput with physiological relevance. However, propelled by automated sorting and microfluidic technologies, the *C. elegans* model has successfully bridged this gap. Emerging platforms—encompassing microfluidic, non-microfluidic, and optofluidic imaging—are advancing rapidly, shifting the field toward a combination of high throughput and single-organism resolution while steadily accelerating imaging speeds (Mahbub et al., 2025). Traditional solid-media nematode culture presents distinct bottlenecks during large-scale applications. The preparation of extensive agar plates prepared with varying drug batches is both labor-intensive and cost-prohibitive, which further complicates the precise control of drug concentrations. Researchers developed fully automated liquid screening workflows compatible with standard 96- to 384-well microplates, resolving previous constraints regarding dosing precision and operational scale (Au - Gao et al., 2019; O'Brien et al., 2025). Because liquid culture systems seamlessly integrate with automated liquid handling robotics, a 384-well plate can be partitioned within minutes. This capacity easily elevates the weekly screening capacity to over 10,000 compounds, enhancing throughput by several orders of magnitude (Mahbub et al., 2025). The advent of microfluidic technology refines the assessment of complex nematode behaviors, overcoming the limitations of traditional microplate assays that struggle to evaluate intricate phenotypes such as chemotaxis, learning, and memory. By executing precise, dynamic control over fluidic and physical environments at the micrometer scale, this technology achieves profound standardization and reproducibility (Pan et al., 2021).

### 4.2 Environmental toxicity assessment

Environmental pollution has become increasingly severe, causing nine million deaths globally each year (Fuller et al., 2022). *C. elegans* exhibits high sensitivity to environmental pollutants, including heavy metals, pesticides, and nanomaterials. These substances disrupt neurodevelopment, induce oxidative stress, and impair synaptic function, resulting in abnormal motor, learning, and sensory behaviors in the nematode (Taylor et al., 2025; Wu J. et al., 2025). Consequently, *C. elegans* is increasingly utilized as a biological sensor to evaluate the neurotoxicity of environmental pollutants, serving as an early warning system for environmental safety and public health (Lemmon et al., 2025). Because heavy metals resist degradation and exhibit significant bioaccumulation, they remain a primary focus of environmental toxicology. Cadmium, a highly toxic heavy metal pollutant, enters the human body through multiple routes, including the consumption of contaminated food and water, or the inhalation of cadmium-containing particles from cigarette smoke and industrial emissions (Arruebarrena et al., 2023). Upon cadmium exposure, *C. elegans* undergoes transcriptomic reprogramming. During this process, expression levels of the heat shock protein gene hsp-16.2 and metallothionein genes (*mtl-1* and *mtl-2*) increase significantly (Cui et al., 2007; Hall et al., 2012; Liu H. et al., 2025). Recent studies identified a novel cadmium-responsive gene, *T08G5.1*, which is dramatically upregulated following exposure. Under identical cadmium exposure conditions, L3 larvae exhibited more extensive transcriptomic reprogramming than L4 larvae. This indicates a heightened transcriptional sensitivity to cadmium pollution during early developmental stages (Almutairi et al., 2024).

### 4.3 Applications of omics technologies

**Figure 3:**
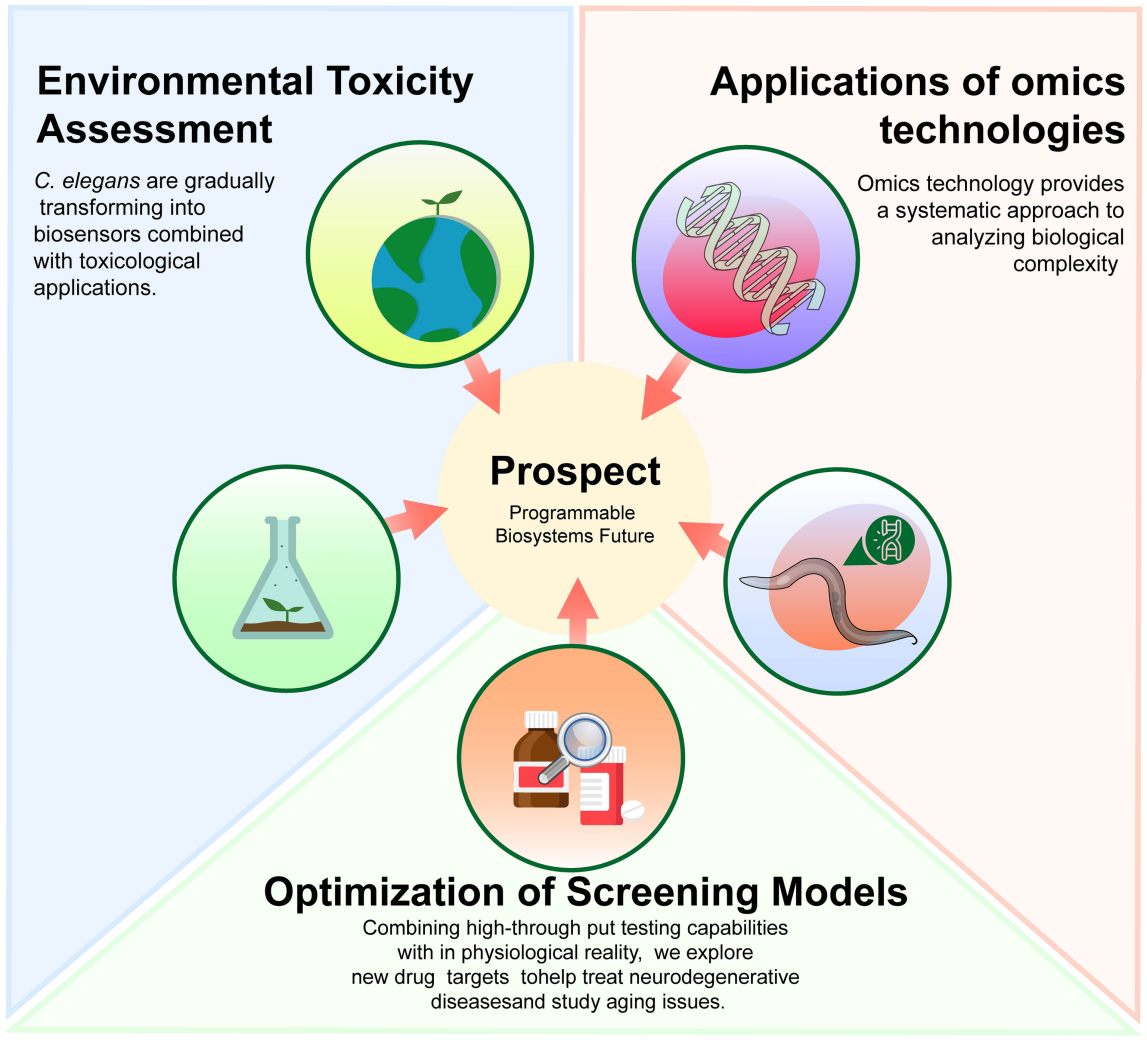
The application prospects of *C. elegans* in neuroscience. Clinical practice with three possible directions for future development as follows: from drug screening to environmental toxicology assessment, as well as the application of omics technology.

High-throughput sequencing technologies are advancing rapidly. Transcriptomic studies in *C. elegans* have transitioned from traditional bulk RNA-seq to a new era of multidimensional single-cell and spatial profiling. Bulk RNA-seq frequently masks transcriptional heterogeneity among individual cells (Ma and Zheng, 2023). By resolving gene expression patterns at single-cell resolution, single-cell RNA sequencing (scRNA-seq) facilitates the construction of high-precision, whole-organism cellular atlases. Cao, Packer, and colleagues demonstrated that scRNA-seq achieves accurate identification of diverse cell types across the nematode. Using pseudotime analysis, they successfully reconstructed complex cellular developmental trajectories (Cao et al., 2017; Packer et al., 2019). Because scRNA-seq requires tissue dissociation, it inevitably strips away cellular spatial coordinates and microenvironmental context. Spatial transcriptomics effectively bridges this technical gap. By integrating high-resolution *in situ* imaging techniques like multiplexed error-robust fluorescence in situ hybridization (MERFISH), researchers can directly map gene expression profiles while preserving the intact anatomical architecture of the nematode (He et al., 2022). The deep integration of single-cell and spatial omics technologies yields a panoramic molecular map essential for deciphering complex physiological mechanisms. Figure 3 outlines the future research directions for *C. elegans*.

## 5 Conclusion

In the fundamental study of molecular biology and brain science, *C. elegans* serves as a uniquely powerful model organism. Despite its established status in fundamental neurobiology, the nematode also presents significant limitations for clinical translation. Electrophysiologically, the organism relies primarily on graded potentials rather than classical action potentials for signal transduction. Pharmacokinetically, its highly impermeable cuticle forms a formidable physical barrier that restricts the delivery of many neuroprotective compounds. Lacking a complex circulatory system and a definitive blood–brain barrier, this model frequently generates false-negative results during *in vivo* drug screening. Finally, behavioral assessments face an inherent ceiling imposed by its extremely streamlined nervous system. While altered locomotion or shortened lifespan adequately reflects baseline neurotoxicity, these metrics fail to authentically recapitulate the complex cognitive decline, memory loss, and higher-order emotional disturbances characteristic of human neurodegenerative diseases.

## References

[ref1] Alirzayeva H. Loureiro R. Koyuncu S. Hommen F. Nabawi Y. Zhang W. H. . (2024). ALS-FUS mutations cause abnormal PARylation and histone H1.2 interaction, leading to pathological changes. Cell Rep. 43:114626. doi: 10.1016/j.celrep.2024.114626, 39167487

[ref2] Almutairi N. Khan N. Harrison-Smith A. Arlt V. M. Stürzenbaum S. R. (2024). Stage-specific exposure of *Caenorhabditis elegans* to cadmium identifies unique transcriptomic response cascades and an uncharacterized cadmium responsive transcript. Metallomics 16:mfae016. doi: 10.1093/mtomcs/mfae016, 38549424 PMC11066929

[ref3] Alvarez J. Alvarez-Illera P. Santo-Domingo J. Fonteriz R. I. Montero M. (2022). Modeling Alzheimer's disease in *Caenorhabditis elegans*. Biomedicine 10:288. doi: 10.3390/biomedicines10020288, 35203497 PMC8869312

[ref4] Anand C. Abdelnour F. Sipes B. Ma D. Maia P. D. Torok J. . (2025). Selective vulnerability and resilience to Alzheimer's disease tauopathy as a function of genes and the connectome. Brain 148, 3679–3693. doi: 10.1093/brain/awaf179, 40631882 PMC12493037

[ref5] Arruebarrena M. A. Hawe C. T. Lee Y. M. Branco R. C. (2023). Mechanisms of cadmium neurotoxicity. Int. J. Mol. Sci. 24:16558. doi: 10.3390/ijms242316558, 38068881 PMC10706630

[ref6] Baskoylu S. N. Chapkis N. Unsal B. Lins J. Schuch K. Simon J. . (2022). Disrupted autophagy and neuronal dysfunction in *C. elegans* knockin models of FUS amyotrophic lateral sclerosis. Cell Rep. 38:110195. doi: 10.1016/j.celrep.2021.110195, 35081350

[ref7] Beets I. Zels S. Vandewyer E. Demeulemeester J. Caers J. Baytemur E. . (2023). System-wide mapping of peptide-GPCR interactions in *C. elegans*. Cell Rep. 42:113058. doi: 10.1016/j.celrep.2023.113058, 37656621 PMC7615250

[ref8] Bhandari A. Seguin A. Rothenfluh A. (2024). Synaptic mechanisms of ethanol tolerance and neuroplasticity: insights from invertebrate models. Int. J. Mol. Sci. 25:6838. doi: 10.3390/ijms25136838, 38999947 PMC11241699

[ref9] Bicca Obetine Baptista F. Arantes L. P. Machado M. L. da Silva A. F. Marafiga Cordeiro L. da Silveira T. L. . (2020). Diphenyl diselenide protects a *Caenorhabditis elegans* model for Huntington's disease by activation of the antioxidant pathway and a decrease in protein aggregation. Metallomics 12, 1142–1158. doi: 10.1039/d0mt00074d, 32453327

[ref10] Blaxter M. Denver D. R. (2012). The worm in the world and the world in the worm. BMC Biol. 10:57. doi: 10.1186/1741-7007-10-57, 22731915 PMC3382423

[ref11] Bodhicharla R. Nagarajan A. Winter J. Adenle A. Nazir A. Brady D. . (2012). Effects of α-synuclein overexpression in transgenic *Caenorhabditis elegans* strains. CNS Neurol. Disord. Drug Targets 11, 965–975. doi: 10.2174/1871527311211080005, 23244416 PMC3744922

[ref12] Brenner S. (1974). The genetics of *Caenorhabditis elegans*. Genetics 77, 71–94. doi: 10.1093/genetics/77.1.71, 4366476 PMC1213120

[ref13] Brignull H. R. Moore F. E. Tang S. J. Morimoto R. I. (2006). Polyglutamine proteins at the pathogenic threshold display neuron-specific aggregation in a pan-neuronal *Caenorhabditis elegans* model. J. Neurosci. 26, 7597–7606. doi: 10.1523/jneurosci.0990-06.2006, 16855087 PMC6674286

[ref14] Brunetti G. Di Rosa G. Scuto M. Leri M. Stefani M. Schmitz-Linneweber C. . (2020). Healthspan maintenance and prevention of Parkinson's-like phenotypes with hydroxytyrosol and oleuropein aglycone in *C. elegans*. Int. J. Mol. Sci. 21:2588. doi: 10.3390/ijms21072588, 32276415 PMC7178172

[ref15] Cabreiro F. Ackerman D. Doonan R. Araiz C. Back P. Papp D. . (2011). Increased life span from overexpression of superoxide dismutase in *Caenorhabditis elegans* is not caused by decreased oxidative damage. Free Radic. Biol. Med. 51, 1575–1582. doi: 10.1016/j.freeradbiomed.2011.07.020, 21839827 PMC3202636

[ref16] Calahorro F. Izquierdo P. G. (2018). The presynaptic machinery at the synapse of *C. elegans*. Invertebr. Neurosci. 18:4. doi: 10.1007/s10158-018-0207-5, 29532181 PMC5851683

[ref17] Caldero-Escudero E. Romero-Sanz S. De la Fuente S. (2024). Using *C. elegans* as a model for neurodegenerative diseases: methodology and evaluation. Methods Cell Biol. 188, 1–34. doi: 10.1016/bs.mcb.2024.03.004, 38880519

[ref18] Cao J. Packer J. S. Ramani V. Cusanovich D. A. Huynh C. Daza R. . (2017). Comprehensive single-cell transcriptional profiling of a multicellular organism. Science 357, 661–667. doi: 10.1126/science.aam8940, 28818938 PMC5894354

[ref19] Carroll T. A. Johnson G. V. Nehrke K. (2026). Tau clearance reverses touch neuron dysfunction in both young and aged *C. elegans*. J. Alzheimer's Dis 109, 189–202. doi: 10.1177/13872877251392935, 41284589 PMC13006792

[ref20] Cell editorial team (2024). The expanding world of neuroscience. Cell 187, 5797–5798. doi: 10.1016/j.cell.2024.09.015, 39423798

[ref21] Chase D. L. Koelle M. R. (2007). Biogenic amine neurotransmitters in *C. elegans*. WormBook. 2007, 1–15. doi: 10.1895/wormbook.1.132.1, 18050501 PMC4781333

[ref22] Chaudhary S. A. Chaudhary S. Rawat S. (2025). Understanding Parkinson's disease: current trends and its multifaceted complications. Front. Aging Neurosci. 17:1617106. doi: 10.3389/fnagi.2025.1617106, 41049533 PMC12488584

[ref23] Chen M. Chen S. Liu K. Ye Z. Qian Y. He J. . (2024). Putative adverse outcome pathway for Parkinson's disease-like symptoms induced by silicon quantum dots based on in vivo/vitro approaches. ACS Nano 18, 25271–25289. doi: 10.1021/acsnano.4c08516, 39186478

[ref24] Chen Z.-Y. Zhang Y. (2022). Animal models of Alzheimer’s disease: applications, evaluation, and perspectives. Zool. Res. 43, 1026–1040. doi: 10.24272/j.issn.2095-8137.2022.289, 36317468 PMC9700500

[ref25] Chen L. Zhang S. Liu S. Gao S. (2024). Amyotrophic lateral sclerosis mechanism: insights from the *Caenorhabditis elegans* models. Cells 13:99. doi: 10.3390/cells13010099, 38201303 PMC10778397

[ref26] Cho C. E. Brueggemann C. L'Etoile N. D. Bargmann C. I. (2016). Parallel encoding of sensory history and behavioral preference during *Caenorhabditis elegans* olfactory learning. eLife 5:e14000. doi: 10.7554/eLife.14000, 27383131 PMC4935464

[ref27] Cisek P. Green A. M. (2024). Toward a neuroscience of natural behavior. Curr. Opin. Neurobiol. 86:102859. doi: 10.1016/j.conb.2024.102859, 38583263

[ref28] Cook S. J. Crouse C. M. Yemini E. Hall D. H. Emmons S. W. Hobert O. (2020). The connectome of the *Caenorhabditis elegans* pharynx. J. Comp. Neurol. 528, 2767–2784. doi: 10.1002/cne.24932, 32352566 PMC7601127

[ref29] Cooper J. F. Van Raamsdonk J. M. (2018). Modeling Parkinson's Disease in *C. elegans*. J. Parkinsons Dis. 8, 17–32. doi: 10.3233/jpd-171258, 29480229 PMC5836411

[ref30] Corsi A. K. Wightman B. Chalfie M. (2015). A transparent window into biology: a primer on *Caenorhabditis elegans*. Genetics 200, 387–407. doi: 10.1534/genetics.115.176099, 26088431 PMC4492366

[ref31] Cui Y. McBride S. J. Boyd W. A. Alper S. Freedman J. H. (2007). Toxicogenomic analysis of *Caenorhabditis elegans* reveals novel genes and pathways involved in the resistance to cadmium toxicity. Genome Biol. 8:R122. doi: 10.1186/gb-2007-8-6-r122, 17592649 PMC2394766

[ref32] Culetto E. Sattelle D. B. (2000). A role for *Caenorhabditis elegans* in understanding the function and interactions of human disease genes. Hum. Mol. Genet. 9, 869–877. doi: 10.1093/hmg/9.6.869, 10767309

[ref33] da Silva L. P. D. da Cruz Guedes E. Fernandes I. C. O. Pedroza L. A. L. da Silva Pereira G. J. Gubert P. (2024). Exploring *Caenorhabditis elegans* as Parkinson's disease model: neurotoxins and genetic implications. Neurotox. Res. 42:11. doi: 10.1007/s12640-024-00686-3, 38319410

[ref34] da Silva A. M. P. da Santos do Nascimento M. Tudella G. C. N. Reis R. D. F. Corin A. S. de Bastos Maximiano M. L. . (2026). Bibliometric analysis of global research trends on the relationship between Alzheimer's disease and type 2 diabetes mellitus. J. Diabetes Metab. Disord. 25:12. doi: 10.1007/s40200-025-01812-4, 41451400 PMC12728145

[ref35] Dag U. Nwabudike I. Kang D. Gomes M. A. Kim J. Atanas A. A. . (2023). Dissecting the functional organization of the *C. elegans* serotonergic system at whole-brain scale. Cell 186, 2574–2592.e20. doi: 10.1016/j.cell.2023.04.023, 37192620 PMC10484565

[ref36] Daigle I. Li C. (1993). Apl-1, a *Caenorhabditis elegans* gene encoding a protein related to the human beta-amyloid protein precursor. Proc. Natl. Acad. Sci. USA 90, 12045–12049. doi: 10.1073/pnas.90.24.12045, 8265668 PMC48122

[ref37] Di Carlo M. (2012). Simple model systems: a challenge for Alzheimer's disease. Immun. Ageing 9, 3. doi: 10.1186/1742-4933-9-3, 22507659 PMC3388466

[ref38] Donnelly J. L. Clark C. M. Leifer A. M. Pirri J. K. Haburcak M. Francis M. M. . (2013). Monoaminergic orchestration of motor programs in a complex *C. elegans* behavior. PLoS Biol. 11:e1001529. doi: 10.1371/journal.pbio.1001529, 23565061 PMC3614513

[ref39] Doonan R. McElwee J. J. Matthijssens F. Walker G. A. Houthoofd K. Back P. . (2008). Against the oxidative damage theory of aging: superoxide dismutases protect against oxidative stress but have little or no effect on life span in *Caenorhabditis elegans*. Genes Dev. 22, 3236–3241. doi: 10.1101/gad.504808, 19056880 PMC2600764

[ref40] Dormann D. Rodde R. Edbauer D. Bentmann E. Fischer I. Hruscha A. . (2010). ALS-associated fused in sarcoma (FUS) mutations disrupt Transportin-mediated nuclear import. EMBO J. 29, 2841–2857. doi: 10.1038/emboj.2010.143, 20606625 PMC2924641

[ref41] Drake J. Link C. D. Butterfield D. A. (2003). Oxidative stress precedes fibrillar deposition of Alzheimer's disease amyloid beta-peptide (1-42) in a transgenic *Caenorhabditis elegans* model. Neurobiol. Aging 24, 415–420. doi: 10.1016/s0197-4580(02)00225-7, 12600717

[ref42] Duerr J. S. McManus J. R. Crowell J. A. Rand J. B. (2021). Analysis of *Caenorhabditis elegans* acetylcholine synthesis mutants reveals a temperature-sensitive requirement for cholinergic neuromuscular function. Genetics 218:iyab078. doi: 10.1093/genetics/iyab078, 34028515 PMC9335933

[ref43] Eck R. J. Stair J. G. Kraemer B. C. Liachko N. F. (2023). Simple models to understand complex disease: 10 years of progress from *Caenorhabditis elegans* models of amyotrophic lateral sclerosis and frontotemporal lobar degeneration. Front. Neurosci. 17:1300705. doi: 10.3389/fnins.2023.1300705, 38239833 PMC10794587

[ref44] El Din R. H. Thabit S. (2024). Quinic acid protects against the development of Huntington's disease in *Caenorhabditis elegans* model. BMC Complement Med Ther 24:377. doi: 10.1186/s12906-024-04670-4, 39468600 PMC11514749

[ref45] Emerson S. Hay M. Smith M. Granger R. Blauch D. Snyder N. . (2021). Acetylcholine signaling genes are required for cocaine-stimulated egg laying in *Caenorhabditis elegans*. G3 (Bethesda) 11:jkab143. doi: 10.1093/g3journal/jkab143, 33914087 PMC8763240

[ref46] Emmons S. W. (2024). Comprehensive analysis of the *C. elegans* connectome reveals novel circuits and functions of previously unstudied neurons. PLoS Biol. 22:e3002939. doi: 10.1371/journal.pbio.3002939, 39689061 PMC11651592

[ref47] Fadda M. De Fruyt N. Borghgraef C. Watteyne J. Peymen K. Vandewyer E. . (2020). NPY/NPF-related neuropeptide FLP-34 signals from serotonergic neurons to modulate aversive olfactory learning in *Caenorhabditis elegans*. J. Neurosci. 40, 6018–6034. doi: 10.1523/jneurosci.2674-19.2020, 32576621 PMC7392509

[ref48] Fang E. F. Hou Y. Palikaras K. Adriaanse B. A. Kerr J. S. Yang B. . (2019). Mitophagy inhibits amyloid-β and tau pathology and reverses cognitive deficits in models of Alzheimer's disease. Nat. Neurosci. 22, 401–412. doi: 10.1038/s41593-018-0332-9, 30742114 PMC6693625

[ref49] Fatouros C. Pir G. J. Biernat J. Koushika S. P. Mandelkow E. Mandelkow E. M. . (2012). Inhibition of tau aggregation in a novel *Caenorhabditis elegans* model of tauopathy mitigates proteotoxicity. Hum. Mol. Genet. 21, 3587–3603. doi: 10.1093/hmg/dds190, 22611162

[ref50] Fay D. S. Fluet A. Johnson C. J. Link C. D. (1998). In vivo aggregation of beta-amyloid peptide variants. J. Neurochem. 71, 1616–1625. doi: 10.1046/j.1471-4159.1998.71041616.x, 9751195

[ref51] Feng L. Marquina-Solis J. Yue L. Harnagel A. Greenfeld Y. Bargmann C. I. (2025). Context-dependent serotonin signaling links dietary quality to foraging decisions. Nat. Commun. 16:10479. doi: 10.1038/s41467-025-65491-8, 41290599 PMC12647731

[ref52] Fernandez R. W. Wei K. Wang E. Y. Mikalauskaite D. Olson A. Pepper J. . (2020). Cellular expression and functional roles of all 26 neurotransmitter GPCRs in the *C. elegans* egg-laying circuit. J. Neurosci. 40, 7475–7488. doi: 10.1523/jneurosci.1357-20.2020, 32847964 PMC7511189

[ref53] Flavell S. W. Pokala N. Macosko E. Z. Albrecht D. R. Larsch J. Bargmann C. I. (2013). Serotonin and the neuropeptide PDF initiate and extend opposing behavioral states in *C. elegans*. Cell 154, 1023–1035. doi: 10.1016/j.cell.2013.08.001, 23972393 PMC3942133

[ref54] Fong S. Teo E. Ng L. F. Chen C. B. Lakshmanan L. N. Tsoi S. Y. . (2016). Energy crisis precedes global metabolic failure in a novel *Caenorhabditis elegans* Alzheimer disease model. Sci. Rep. 6:33781. doi: 10.1038/srep33781, 27653553 PMC5031964

[ref55] Frankel E. B. Kurshan P. T. (2025). Principles of synaptogenesis: insights from Caenorhabditiselegans. Curr. Opin. Neurobiol. 93:103056. doi: 10.1016/j.conb.2025.103056, 40483740

[ref56] Fuller R. Landrigan P. J. Balakrishnan K. Bathan G. Bose-O'Reilly S. Brauer M. . (2022). Pollution and health: a progress update. Lancet Planet. Heath 6, e535–e547. doi: 10.1016/S2542-5196(22)00090-0, 35594895 PMC11995256

[ref57] Gaeta A. L. Willicott K. Willicott C. W. McKay L. E. Keogh C. M. Altman T. J. . (2023). Mechanistic impacts of bacterial diet on dopaminergic neurodegeneration in a *Caenorhabditis elegans* α-synuclein model of Parkinson's disease. iScience 26:106859. doi: 10.1016/j.isci.2023.106859, 37260751 PMC10227375

[ref58] Gao S. Chen W. Zhang N. Xu C. Jing H. Zhang W. . (2019). A high-throughput assay for the prediction of chemical toxicity by automated phenotypic profiling of *Caenorhabditis elegans*. JoVE 145:e59082. doi: 10.3791/59082 30933063

[ref59] Garcia-Toscano L. Currey H. N. Hincks J. C. Stair J. G. Lehrbach N. J. Liachko N. F. (2024). Decreased Hsp90 activity protects against TDP-43 neurotoxicity in a *C. elegans* model of amyotrophic lateral sclerosis. PLoS Genet. 20:e1011518. doi: 10.1371/journal.pgen.1011518, 39724103 PMC11709271

[ref60] Gidalevitz T. Krupinski T. Garcia S. Morimoto R. I. (2009). Destabilizing protein polymorphisms in the genetic background direct phenotypic expression of mutant SOD1 toxicity. PLoS Genet. 5:e1000399. doi: 10.1371/journal.pgen.1000399, 19266020 PMC2642731

[ref61] Griffin E. F. Owens M. G. (2025). Dopaminergic neurodegeneration in *C. elegans* cultivated with *Porphorymonas gingivalis*. MicroPubl. Biol. 2025:001423. doi: 10.17912/micropub.biology.001423, 39839711 PMC11749262

[ref62] Gu Y. Wang M. Mao Y. (2026). The microbiota-gut-brain axis in Huntington’s disease: evidence, mechanisms and therapeutic opportunities. Front. Neuroendocrinol. 81:101247. doi: 10.1016/j.yfrne.2026.101247, 41876029

[ref63] Hall J. Haas K. L. Freedman J. H. (2012). Role of MTL-1, MTL-2, and CDR-1 in mediating cadmium sensitivity in *Caenorhabditis elegans*. Toxicol. Sci. 128, 418–426. doi: 10.1093/toxsci/kfs166, 22552775 PMC3493192

[ref64] Han B. Dong Y. Zhang L. Liu Y. Rabinowitch I. Bai J. (2017). Dopamine signaling tunes spatial pattern selectivity in *C. elegans*. eLife 6:e22896. doi: 10.7554/eLife.22896, 28349862 PMC5370180

[ref65] Hardy J. Selkoe D. J. (2002). The amyloid hypothesis of Alzheimer's disease: progress and problems on the road to therapeutics. Science 297, 353–356. doi: 10.1126/science.1072994, 12130773

[ref66] Harris T. W. Chen N. Cunningham F. Tello-Ruiz M. Antoshechkin I. Bastiani C. . (2004). WormBase: a multi-species resource for nematode biology and genomics. Nucleic Acids Res. 32, 411D–4417D. doi: 10.1093/nar/gkh066, 14681445 PMC308800

[ref67] He S. Bhatt R. Brown C. Brown E. A. Buhr D. L. Chantranuvatana K. . (2022). High-plex imaging of RNA and proteins at subcellular resolution in fixed tissue by spatial molecular imaging. Nat. Biotechnol. 40, 1794–1806. doi: 10.1038/s41587-022-01483-z, 36203011

[ref68] Hendi A. Kurashina M. Mizumoto K. (2019). Intrinsic and extrinsic mechanisms of synapse formation and specificity in *C. elegans*. Cell. Mol. Life Sci. 76, 2719–2738. doi: 10.1007/s00018-019-03109-1, 31037336 PMC11105629

[ref69] Huang J. Chen S. Hu L. Niu H. Sun Q. Li W. . (2018). Mitoferrin-1 is involved in the progression of Alzheimer's disease through targeting mitochondrial Iron metabolism in a *Caenorhabditis elegans* model of Alzheimer's disease. Neuroscience 385, 90–101. doi: 10.1016/j.neuroscience.2018.06.011, 29908215

[ref70] Jan L. Y. (2018). Neuroscience: Past and Future. Neuron 98, 10–11. doi: 10.1016/j.neuron.2018.03.029, 29621483

[ref71] Jin Y. (2015). Unraveling the mechanisms of synapse formation and axon regeneration: the awesome power of *C. elegans* genetics. Sci. China Life Sci. 58, 1084–1088. doi: 10.1007/s11427-015-4962-9, 26563175 PMC4696538

[ref72] Källstig E. McCabe B. D. Schneider B. L. (2021). The links between ALS and NF-κB. Int. J. Mol. Sci. 22:3875. doi: 10.3390/ijms22083875, 33918092 PMC8070122

[ref73] Katz M. Corson F. Keil W. Singhal A. Bae A. Lu Y. . (2019). Glutamate spillover in *C. elegans* triggers repetitive behavior through presynaptic activation of MGL-2/mGluR5. Nat. Commun. 10:1882. doi: 10.1038/s41467-019-09581-4, 31015396 PMC6478929

[ref74] Katz M. Robertson T. Ngo S. T. Yarlagadda S. Henderson R. D. McCombe P. A. . (2026). Review of the pathology of muscle in amyotrophic lateral sclerosis. Int. J. Mol. Sci. 27:2802. doi: 10.3390/ijms27062802, 41898662 PMC13026879

[ref75] Kindt K. S. Quast K. B. Giles A. C. De S. Hendrey D. Nicastro I. . (2007). Dopamine mediates context-dependent modulation of sensory plasticity in *C. elegans*. Neuron 55, 662–676. doi: 10.1016/j.neuron.2007.07.023, 17698017

[ref76] Kow R. L. Black A. H. Henderson B. P. Kraemer B. C. (2023). Sut-6/NIPP1 modulates tau toxicity. Hum. Mol. Genet. 32, 2292–2306. doi: 10.1093/hmg/ddad049, 37000013 PMC10321383

[ref77] Kraemer B. C. Zhang B. Leverenz J. B. Thomas J. H. Trojanowski J. Q. Schellenberg G. D. (2003). Neurodegeneration and defective neurotransmission in a *Caenorhabditis elegans* model of tauopathy. Proc. Natl. Acad. Sci. USA 100, 9980–9985. doi: 10.1073/pnas.1533448100, 12872001 PMC187908

[ref78] Kwaśniewska K. Fic W. Polak-Szczybyło E. (2026). Vitamins as modulators of neurodegenerative disease pathways: mechanisms and therapeutic perspectives. Nutrients 18:995. doi: 10.3390/nu18060995, 41901170 PMC13028732

[ref79] Lagier-Tourenne C. Cleveland D. W. (2009). Rethinking ALS: the FUS about TDP-43. Cell 136, 1001–1004. doi: 10.1016/j.cell.2009.03.006, 19303844 PMC3110083

[ref80] Lapierre L. R. Hansen M. (2012). Lessons from *C. elegans*: signaling pathways for longevity. Trends Endocrinol. Metab. 23, 637–644. doi: 10.1016/j.tem.2012.07.007, 22939742 PMC3502657

[ref81] Lee H. J. Alirzayeva H. Koyuncu S. Rueber A. Noormohammadi A. Vilchez D. (2023). Cold temperature extends longevity and prevents disease-related protein aggregation through PA28γ-induced proteasomes. Nat. Aging 3, 546–566. doi: 10.1038/s43587-023-00383-4, 37118550 PMC10191861

[ref82] Lee A. L. Ung H. M. Sands L. P. Kikis E. A. (2017). A new *Caenorhabditis elegans* model of human huntingtin 513 aggregation and toxicity in body wall muscles. PLoS One 12:e0173644. doi: 10.1371/journal.pone.0173644, 28282438 PMC5345860

[ref83] Lemmon D. Lopez G. Schiffbauer J. Sensale S. Sun G. (2025). Harnessing *C. elegans* as a biosensor: integrating microfluidics, image analysis, and machine learning for environmental sensing. Sensors (Basel) 25:6570. doi: 10.3390/s25216570, 41228792 PMC12610099

[ref84] Li C. Kim K. (2014). Family of FLP peptides in Caenorhabditis elegans and related nematodes. Front. Endocrinol. (Lausanne) 5:150. doi: 10.3389/fendo.2014.00150, 25352828 PMC4196577

[ref85] Liachko N. F. Guthrie C. R. Kraemer B. C. (2010). Phosphorylation promotes neurotoxicity in a *Caenorhabditis elegans* model of TDP-43 proteinopathy. J. Neurosci. 30, 16208–16219. doi: 10.1523/jneurosci.2911-10.2010, 21123567 PMC3075589

[ref86] Liang Y. Zhou Y. Zhou C. Cai X. Liu L. Wei F. . (2024). Sertraline promotes health and longevity in *Caenorhabditis elegans*. Gerontology 70, 408–417. doi: 10.1159/000536227 38228128

[ref87] Limke A. Scharpf I. Blesing F. von Mikecz A. (2023). Tire components, age and temperature accelerate neurodegeneration in *C. elegans* models of Alzheimer's and Parkinson's disease. Environ. Pollut. 328:121660. doi: 10.1016/j.envpol.2023.121660, 37080524

[ref88] Lin H. Gao Y. Zhang C. Ma B. Wu M. Cui X. . (2022). Autophagy regulation influences β-amyloid toxicity in transgenic *Caenorhabditis elegans*. Front. Aging Neurosci. 14:885145. doi: 10.3389/fnagi.2022.885145, 35645788 PMC9133694

[ref89] Link C. D. (1995). Expression of human beta-amyloid peptide in transgenic *Caenorhabditis elegans*. Proc. Natl. Acad. Sci. USA 92, 9368–9372. doi: 10.1073/pnas.92.20.9368, 7568134 PMC40986

[ref90] Link C. D. Fonte V. Roberts C. M. Hiester B. Silverman M. A. Stein G. H. (2008). The beta amyloid peptide can act as a modular aggregation domain. Neurobiol. Dis. 32, 420–425. doi: 10.1016/j.nbd.2008.08.003, 18778773 PMC2646107

[ref91] Liu H. Earley B. Mendoza A. Hunt P. Teng S. Schneider D. L. . (2025). A single high-zinc activation enhancer can control two genes oriented head-to-head in *Caenorhabditis elegans*. G3 (Bethesda) 15:jkaf089. doi: 10.1093/g3journal/jkaf089, 40408183 PMC12239621

[ref92] Liu L. Hao X. Bai Y. Tian Y. (2025). The soil Mycobacterium sp. promotes health and longevity through different bacteria-derived molecules in *Caenorhabditis elegans*. Aging Cell 24:e14416. doi: 10.1111/acel.14416, 39560153 PMC11896450

[ref93] Liu J. Kulkarni A. Gao Y. Q. Urul D. A. Hamelin R. Novotny B. . (2024). Organ-specific electrophile responsivity mapping in live *C. elegans*. Cell 187, 7450–7469.e29. doi: 10.1016/j.cell.2024.10.014, 39504959

[ref94] Liu H. Zheng P. Wang X. (2026). From small brains to smart machines: translating *Caenorhabditis elegans* neural circuits into artificial intelligence. Front. Neural Circuits 20:1731513. doi: 10.3389/fncir.2026.1731513, 41878287 PMC13006640

[ref95] Liu Y. Zhi D. Wang X. Fei D. Zhang Z. Wu Z. . (2018). Kushui rose (R. Setate x *R. rugosa*) decoction exerts antitumor effects in *C. elegans* by downregulating Ras/MAPK pathway and resisting oxidative stress. Int. J. Mol. Med. 42, 1411–1417. doi: 10.3892/ijmm.2018.3738, 29956725 PMC6089776

[ref96] Luo Y. Wu Y. Brown M. Link C. D. (2009). *Caenorhabditis elegans* model for initial screening and mechanistic evaluation of potential new drugs for aging and Alzheimer’s disease," in Methods of Behavior Analysis in Neuroscience, ed. Buccafusco J.J.. (Boca Raton (FL): CRC Press/Taylor & Francis), 311–327. 21204333

[ref98] Ma F. Zheng C. (2023). Transcriptome age of individual cell types in *Caenorhabditis elegans*. Proc. Natl. Acad. Sci. USA 120:e2216351120. doi: 10.1073/pnas.2216351120, 36812209 PMC9992843

[ref99] Mahbub T. B. Safaeian P. Sohrabi S. (2025). Automated platforms in *C. elegans* research: integration of microfluidics, robotics, and artificial intelligence. Micromachines 16:1138. doi: 10.3390/mi16101138, 41156385 PMC12566038

[ref101] Mandelkow E. M. Mandelkow E. (2012). Biochemistry and cell biology of tau protein in neurofibrillary degeneration. Cold Spring Harb. Perspect. Med. 2:a006247. doi: 10.1101/cshperspect.a006247, 22762014 PMC3385935

[ref102] Maro G. S. Gao S. Olechwier A. M. Hung W. L. Liu M. Özkan E. . (2015). MADD-4/Punctin and Neurexin Organize *C. elegans* GABAergic Postsynapses through Neuroligin. Neuron 86, 1420–1432. doi: 10.1016/j.neuron.2015.05.015, 26028574 PMC4672740

[ref103] Maulik M. Mitra S. Bult-Ito A. Taylor B. E. Vayndorf E. M. (2017). Behavioral phenotyping and pathological indicators of Parkinson's disease in *C. elegans* models. Front. Genet. 8:77. doi: 10.3389/fgene.2017.00077, 28659967 PMC5468440

[ref104] McColl G. Roberts B. R. Gunn A. P. Perez K. A. Tew D. J. Masters C. L. . (2009). The *Caenorhabditis elegans* a beta 1-42 model of Alzheimer disease predominantly expresses a beta 3-42. J. Biol. Chem. 284, 22697–22702. doi: 10.1074/jbc.C109.028514, 19574211 PMC2755678

[ref105] McColl G. Roberts B. R. Pukala T. L. Kenche V. B. Roberts C. M. Link C. D. . (2012). Utility of an improved model of amyloid-beta (aβ₁₋₄₂) toxicity in *Caenorhabditis elegans* for drug screening for Alzheimer's disease. Mol. Neurodegener. 7:57. doi: 10.1186/1750-1326-7-57, 23171715 PMC3519830

[ref106] McDermott J. B. Aamodt S. Aamodt E. (1996). Ptl-1, a *Caenorhabditis elegans* gene whose products are homologous to the tau microtubule-associated proteins. Biochemistry 35, 9415–9423. doi: 10.1021/bi952646n, 8755720

[ref107] Meijboom K. E. Brown R. H. (2022). Approaches to gene modulation therapy for ALS. Neurotherapeutics 19, 1159–1179. doi: 10.1007/s13311-022-01285-w, 36068427 PMC9587165

[ref108] Menge S. Decker L. Freischmidt A. (2025). Genetics of ALS - genes and modifier. Curr. Opin. Neurol. 38, 568–573. doi: 10.1097/wco.0000000000001416, 40772638 PMC12419016

[ref109] Millet J. R. M. Faumont S. Schatz A. B. White A. M. Chicas-Cruz K. D. Lockery S. R. (2025). *C. elegans* food choice exhibits effort discounting-like behavior. eLife 14:e106792. doi: 10.7554/eLife.106792, 41324250 PMC12668669

[ref110] Mizumoto K. Jin Y. Bessereau J. L. (2023). Synaptogenesis: unmasking molecular mechanisms using *Caenorhabditis elegans*. Genetics 223:iyac176. doi: 10.1093/genetics/iyac176, 36630525 PMC9910414

[ref111] Morley J. F. Brignull H. R. Weyers J. J. Morimoto R. I. (2002). The threshold for polyglutamine-expansion protein aggregation and cellular toxicity is dynamic and influenced by aging in *Caenorhabditis elegans*. Proc. Natl. Acad. Sci. USA 99, 10417–10422. doi: 10.1073/pnas.152161099, 12122205 PMC124929

[ref112] Murakami T. Yang S. P. Xie L. Kawano T. Fu D. Mukai A. . (2012). ALS mutations in FUS cause neuronal dysfunction and death in *Caenorhabditis elegans* by a dominant gain-of-function mechanism. Hum. Mol. Genet. 21, 1–9. doi: 10.1093/hmg/ddr417, 21949354 PMC3235006

[ref113] Muralidhara I. Hardege I. (2025). The dopaminergic system of *Caenorhabditis elegans*. R. Soc. Open Sci. 12:250843. doi: 10.1098/rsos.250843, 41127805 PMC12539964

[ref114] Nass R. Hall D. H. Miller D. M. 3rd Blakely R. D. (2002). Neurotoxin-induced degeneration of dopamine neurons in *Caenorhabditis elegans*. Proc. Natl. Acad. Sci. USA 99, 3264–3269. doi: 10.1073/pnas.042497999, 11867711 PMC122507

[ref115] Natale C. Barzago M. M. Diomede L. (2020). *Caenorhabditis elegans* models to investigate the mechanisms underlying tau toxicity in Tauopathies. Brain Sci. 10:838. doi: 10.3390/brainsci10110838, 33187241 PMC7697895

[ref116] Navarro-Hortal M. D. Romero-Márquez J. M. Osta S. Jiménez-Trigo V. Muñoz-Ollero P. Varela-López A. (2022). Natural bioactive products and Alzheimer's disease pathology: lessons from *Caenorhabditis elegans* transgenic models. Diseases 10:51. doi: 10.3390/diseases10020028, 35645249 PMC9149938

[ref117] Nelson M. D. Lee K. H. Churgin M. A. Hill A. J. Van Buskirk C. Fang-Yen C. . (2014). FMRFamide-like FLP-13 neuropeptides promote quiescence following heat stress in *Caenorhabditis elegans*. Curr. Biol. 24, 2406–2410. doi: 10.1016/j.cub.2014.08.037, 25264253 PMC4254296

[ref118] Nicosia V. Vértes P. E. Schafer W. R. Latora V. Bullmore E. T. (2013). Phase transition in the economically modeled growth of a cellular nervous system. Proc. Natl. Acad. Sci. USA 110, 7880–7885. doi: 10.1073/pnas.1300753110, 23610428 PMC3651470

[ref119] Nusbaum M. P. Blitz D. M. Marder E. (2017). Functional consequences of neuropeptide and small-molecule co-transmission. Nat. Rev. Neurosci. 18, 389–403. doi: 10.1038/nrn.2017.56, 28592905 PMC5547741

[ref120] O'Brien T. J. Barlow I. L. Feriani L. Brown A. E. X. (2025). High-throughput tracking enables systematic phenotyping and drug repurposing in *C. elegans* disease models. eLife 12:e92491. doi: 10.7554/eLife.92491, 39773880 PMC11709427

[ref121] O'Neill K. Shaw R. Bolger I. Tam O. H. Phatnani H. Gale Hammell M. (2025). ALS molecular subtypes are a combination of cellular and pathological features learned by deep multiomics classifiers. Cell Rep. 44:115402. doi: 10.1016/j.celrep.2025.115402, 40067829 PMC12011103

[ref122] Packer J. S. Zhu Q. Huynh C. Sivaramakrishnan P. Preston E. Dueck H. . (2019). A lineage-resolved molecular atlas of *C. elegans* embryogenesis at single-cell resolution. Science 365:6459. doi: 10.1126/science.aax1971, 31488706 PMC7428862

[ref123] Pan P. Song P. Dong X. Zhang W. Sun Y. Liu X. (2021). “9 - microfluidic devices for immobilization and micromanipulation of single cells and small organisms,” in Microfluidic devices for biomedical applications (second edition), eds. Li X. Zhou Y. (Cambridge: Woodhead publishing), 391–412.

[ref124] Panda M. Fakitsa M. Markaki M. Tavernarakis N. (2025). *Caenorhabditis elegans* as an emerging high throughput chronotherapeutic drug screening platform for human neurodegenerative disorders. Adv. Drug Deliv. Rev. 224:115655. doi: 10.1016/j.addr.2025.115655, 40683385

[ref125] Papaioannou S. Holden-Dye L. Walker R. J. (2008). The actions of *Caenorhabditis elegans* neuropeptide-like peptides (NLPs) on body wall muscle of Ascaris suum and pharyngeal muscle of *C. elegans*. Acta Biol. Hung. 59 Suppl, 189–197. doi: 10.1556/ABiol.59.2008.Suppl.28, 18652392

[ref126] Peng J. Y. Liu X. Zeng X. T. Hao Y. Zhang J. H. Li Q. . (2023). Early pheromone perception remodels neurodevelopment and accelerates neurodegeneration in adult *C. elegans*. Cell Rep. 42:112598. doi: 10.1016/j.celrep.2023.112598, 37289584

[ref127] Pir G. J. Buddenkotte J. Alam M. A. Own A. Eck R. J. Kraemer B. C. . (2026). TDP-43 proteinopathies and neurodegeneration: insights from *Caenorhabditis elegans* models. FEBS J. 293, 348–384. doi: 10.1111/febs.70239, 40891506 PMC12820612

[ref128] Pir G. J. Choudhary B. Mandelkow E. (2017). *Caenorhabditis elegans* models of tauopathy. FASEB J. 31, 5137–5148. doi: 10.1096/fj.201701007, 29191965

[ref129] Pir G. J. Choudhary B. Mandelkow E. Mandelkow E. M. (2016). Tau mutant A152T, a risk factor for FTD/PSP, induces neuronal dysfunction and reduced lifespan independently of aggregation in a *C. elegans* Tauopathy model. Mol. Neurodegener. 11:33. doi: 10.1186/s13024-016-0096-1, 27118310 PMC4847334

[ref130] Portman D. S. Bainbridge C. Ward Z. C. Zhang J. (2026). Sexual dimorphism in the nervous system: three principles from the nematode C.Elegans. Curr. Opin. Neurobiol. 97:103175. doi: 10.1016/j.conb.2026.103175, 41724029 PMC13170803

[ref131] Prova N. S. Elsayyid M. W. Tanis J. E. (2026). Superoxide dismutase impacts extracellular vesicle shedding and uptake. Free Radic. Biol. Med. 247, 540–550. doi: 10.1016/j.freeradbiomed.2026.02.008, 41672113 PMC13202618

[ref132] Redl M. Weisenburger S. Wasilewicz A. Grienke U. Lehner M. D. Bredenbröker D. . (2025). Miquelianin and spiraeoside from *Filipendula ulmaria* mitigate α-synuclein accumulation in C.Elegans and reduce the expression of neuroinflammatory cytokines in human microglia. Front. Pharmacol. 16:1720314. doi: 10.3389/fphar.2025.1720314, 41756116 PMC12933199

[ref133] Redweik G. Xue D. (2025). Secreted autotransporter toxin produced by probiotic *Escherichia coli* Nissle 1917 enhances neurodegeneration in *Caenorhabditis elegans*. MicroPubl Biol 2025:001366. doi: 10.17912/micropub.biology.001366, 39950089 PMC11822469

[ref134] Riordan R. Saxton A. Han M. McMillan P. J. Kow R. L. Liachko N. F. . (2025). TMEM106B C-terminal fragments aggregate and drive neurodegenerative proteinopathy in transgenic *Caenorhabditis elegans*. Alzheimers Dement. 21:e14468. doi: 10.1002/alz.14468, 39711302 PMC11848199

[ref135] Ripoll-Sánchez L. Watteyne J. Sun H. Fernandez R. Taylor S. R. Weinreb A. . (2023). The neuropeptidergic connectome of *C. elegans*. Neuron 111, 3570–3589.e5. doi: 10.1016/j.neuron.2023.09.043, 37935195 PMC7615469

[ref136] Rosikon K. D. Bone M. C. Lawal H. O. (2023). Regulation and modulation of biogenic amine neurotransmission in Drosophila and *Caenorhabditis elegans*. Front. Physiol. 14:970405. doi: 10.3389/fphys.2023.970405, 36875033 PMC9978017

[ref137] Roussos A. Kitopoulou K. Borbolis F. Palikaras K. (2023). *Caenorhabditis elegans* as a model system to study human neurodegenerative disorders. Biomolecules 13:478. doi: 10.3390/biom13030478, 36979413 PMC10046667

[ref138] Rubin G. M. Yandell M. D. Wortman J. R. Gabor Miklos G. L. Nelson C. R. Hariharan I. K. . (2000). Comparative genomics of the eukaryotes. Science 287, 2204–2215. doi: 10.1126/science.287.5461.2204, 10731134 PMC2754258

[ref139] Salinas G. Risi G. (2018). *Caenorhabditis elegans*: nature and nurture gift to nematode parasitologists. Parasitology 145, 979–987. doi: 10.1017/s0031182017002165, 29208057

[ref140] Sandhof C. A. Hoppe S. O. Tittelmeier J. Nussbaum-Krammer C. (2020). *C. elegans* models to study the propagation of prions and prion-like proteins. Biomolecules 10:1188. doi: 10.3390/biom10081188, 32824215 PMC7464663

[ref141] Sandhof C. A. Martin N. Tittelmeier J. Schlueter A. Pezzali M. Schoendorf D. C. . (2025). A novel *C. elegans* model for MAPT/tau spreading reveals genes critical for endolysosomal integrity and seeded MAPT/tau aggregation. Autophagy 21, 2963–2981. doi: 10.1080/15548627.2025.2551676, 40851193 PMC12758218

[ref142] Sato H. Kunitomo H. Fei X. Hashimoto K. Iino Y. (2021). Glutamate signaling from a single sensory neuron mediates experience-dependent bidirectional behavior in *Caenorhabditis elegans*. Cell Rep. 35:109177. doi: 10.1016/j.celrep.2021.109177, 34038738

[ref143] Scahill R. I. Farag M. Murphy M. J. Hobbs N. Z. Leocadi M. Langley C. . (2025). Somatic CAG repeat expansion in blood associates with biomarkers of neurodegeneration in Huntington's disease decades before clinical motor diagnosis. Nat. Med. 31, 807–818. doi: 10.1038/s41591-024-03424-6, 39825149 PMC11922752

[ref144] Scheltens P. De Strooper B. Kivipelto M. Holstege H. Chételat G. Teunissen C. E. . (2021). Alzheimer's disease. Lancet 397, 1577–1590. doi: 10.1016/s0140-6736(20)32205-4, 33667416 PMC8354300

[ref145] SenGupta T. Palikaras K. Esbensen Y. Q. Konstantinidis G. Galindo F. J. N. Achanta K. . (2021). Base excision repair causes age-dependent accumulation of single-stranded DNA breaks that contribute to Parkinson disease pathology. Cell Rep. 36:109668. doi: 10.1016/j.celrep.2021.109668, 34496255 PMC8441048

[ref146] Shang H. Wang Z. Sun Y. Zuo C. Wang M. Zheng K. . (2025). Metformin inhibits microglial activation-mediated Cuproptosis by modulating the TLR4/Myd88/NF-κB signaling pathway in Parkinson's disease. Mol. Neurobiol. 63:95. doi: 10.1007/s12035-025-05499-9, 41261257 PMC12630286

[ref147] Sillapakong P. Wakabayashi T. Suzuki K. (2025). Naturido alleviates amyloid β1-42-induced adverse effects in a transgenic *Caenorhabditis elegans* model of Alzheimer's disease. PLoS One 20:e0320636. doi: 10.1371/journal.pone.0320636, 40138382 PMC11940425

[ref148] Sohrabi S. Mor D. E. Kaletsky R. Keyes W. Murphy C. T. (2021). High-throughput behavioral screen in *C. elegans* reveals Parkinson's disease drug candidates. Commun Biol 4:203. doi: 10.1038/s42003-021-01731-z, 33589689 PMC7884385

[ref149] Tao J. Ma Y. C. Yang Z. S. Zou C. G. Zhang K. Q. (2016). Octopamine connects nutrient cues to lipid metabolism upon nutrient deprivation. Sci. Adv. 2:e1501372. doi: 10.1126/sciadv.1501372, 27386520 PMC4928904

[ref150] Taylor S. K. B. Minhas M. H. Tong J. Selvaganapathy P. R. Mishra R. K. Gupta B. P. (2025). Heavy metal exposure disrupts Electrotactic behavior in & Caenorhabditis elegans</em&gt. bioRxiv. doi: 10.1101/2025.05.20.655158

[ref151] Taylor S. R. Santpere G. Weinreb A. Barrett A. Reilly M. B. Xu C. . (2021). Molecular topography of an entire nervous system. Cell 184, 4329–4347.e23. doi: 10.1016/j.cell.2021.06.023, 34237253 PMC8710130

[ref152] Tefera T. W. Steyn F. J. Ngo S. T. Borges K. (2021). CNS glucose metabolism in amyotrophic lateral sclerosis: a therapeutic target? Cell Biosci. 11:14. doi: 10.1186/s13578-020-00511-2, 33431046 PMC7798275

[ref153] Timofeeva A. M. Aulova K. S. Nevinsky G. A. (2025). Modeling Alzheimer's disease: a review of gene-modified and induced animal models, complex cell culture models, and computational modeling. Brain Sci. 15:486. doi: 10.3390/brainsci15050486, 40426657 PMC12109626

[ref154] Tiwari S. Atluri V. Kaushik A. Yndart A. Nair M. (2019). Alzheimer's disease: pathogenesis, diagnostics, and therapeutics. Int. J. Nanomedicine 14, 5541–5554. doi: 10.2147/ijn.S200490, 31410002 PMC6650620

[ref155] Tiwari V. Buvarp E. Borbolis F. Puligilla C. Croteau D. L. Palikaras K. . (2024). Loss of DNA glycosylases improves health and cognitive function in a *C. elegans* model of human tauopathy. Nucleic Acids Res. 52, 10965–10985. doi: 10.1093/nar/gkae705, 39149885 PMC11472166

[ref156] Tomioka M. Jang M. S. Iino Y. (2022). DAF-2c signaling promotes taste avoidance after starvation in *Caenorhabditis elegans* by controlling distinct phospholipase C isozymes. Commun Biol 5:30. doi: 10.1038/s42003-021-02956-8, 35017611 PMC8752840

[ref157] Torres A. K. Mira R. G. Pinto C. Inestrosa N. C. (2025). Studying the mechanisms of neurodegeneration: *C. elegans* advantages and opportunities. Front. Cell. Neurosci. 19:1559151. doi: 10.3389/fncel.2025.1559151, 40207239 PMC11979225

[ref158] Tossing G. Livernoche R. Maios C. Bretonneau C. Labarre A. Parker J. A. (2022). Genetic and pharmacological PARP inhibition reduces axonal degeneration in *C. elegans* models of ALS. Hum. Mol. Genet. 31, 3313–3324. doi: 10.1093/hmg/ddac116, 35594544

[ref159] Tu H. Pinan-Lucarré B. Ji T. Jospin M. Bessereau J. L. (2015). *C. elegans* Punctin clusters GABA(a) receptors via Neuroligin binding and UNC-40/DCC recruitment. Neuron 86, 1407–1419. doi: 10.1016/j.neuron.2015.05.013, 26028575

[ref160] Vaccaro A. Tauffenberger A. Aggad D. Rouleau G. Drapeau P. Parker J. A. (2012). Mutant TDP-43 and FUS cause age-dependent paralysis and neurodegeneration in *C. elegans*. PLoS One 7:e31321. doi: 10.1371/journal.pone.0031321, 22363618 PMC3283630

[ref161] van Ham T. J. Thijssen K. L. Breitling R. Hofstra R. M. Plasterk R. H. Nollen E. A. (2008). *C. elegans* model identifies genetic modifiers of alpha-synuclein inclusion formation during aging. PLoS Genet. 4:e1000027. doi: 10.1371/journal.pgen.1000027, 18369446 PMC2265412

[ref162] Vance C. Rogelj B. Hortobágyi T. De Vos K. J. Nishimura A. L. Sreedharan J. . (2009). Mutations in FUS, an RNA processing protein, cause familial amyotrophic lateral sclerosis type 6. Science 323, 1208–1211. doi: 10.1126/science.1165942, 19251628 PMC4516382

[ref163] Varshney L. R. Chen B. L. Paniagua E. Hall D. H. Chklovskii D. B. (2011). Structural properties of the *Caenorhabditis elegans* neuronal network. PLoS Comput. Biol. 7:e1001066. doi: 10.1371/journal.pcbi.1001066, 21304930 PMC3033362

[ref164] Vela M. García-Gimeno M. A. Sanchis A. Bono-Yagüe J. Cumella J. Lagartera L. . (2022). Neuroprotective effect of IND1316, an indole-based AMPK activator, in animal models of Huntington disease. ACS Chem. Neurosci. 13, 275–287. doi: 10.1021/acschemneuro.1c00758, 34962383 PMC8822144

[ref165] Wang Y. Mandelkow E. (2016). Tau in physiology and pathology. Nat. Rev. Neurosci. 17, 22–35. doi: 10.1038/nrn.2015.1, 26631930

[ref166] Wang X. Mizuguchi K. Hashimoto K. (2026). Excitatory GABA receptors shape locomotor circuit organization in *C. elegans*. Sci. Rep. 16:9407. doi: 10.1038/s41598-026-39358-x, 41703147 PMC13003095

[ref167] Wang C. Vidal B. Sural S. Loer C. Aguilar G. R. Merritt D. M. . (2024). A neurotransmitter atlas of *C. elegans* males and hermaphrodites. eLife 13:e95402. doi: 10.7554/eLife.95402, 39422452 PMC11488851

[ref168] Watteyne J. Chudinova A. Ripoll-Sánchez L. Schafer W. R. Beets I. (2024). Neuropeptide signaling network of *Caenorhabditis elegans*: from structure to behavior. Genetics 228:iyae141. doi: 10.1093/genetics/iyae141, 39344922 PMC11538413

[ref169] Wen Y. P. Fu H. J. Chen Q. Lan C. Qin D. L. Wu J. M. . (2024). Exploring the therapeutic potential of *Nelumbo nucifera* leaf extract against amyloid-beta-induced toxicity in the *Caenorhabditis elegans* model of Alzheimer's disease. Front. Pharmacol. 15:1408031. doi: 10.3389/fphar.2024.1408031, 38983916 PMC11232431

[ref170] White J. G. Southgate E. Thomson J. N. Brenner S. (1986). The structure of the nervous system of the nematode *Caenorhabditis elegans*. Philos. Trans. R. Soc. Lond. Ser. B Biol. Sci. 314, 1–340. doi: 10.1098/rstb.1986.0056, 22462104

[ref171] Witvliet D. Mulcahy B. Mitchell J. K. Meirovitch Y. Berger D. R. Wu Y. . (2021). Connectomes across development reveal principles of brain maturation. Nature 596, 257–261. doi: 10.1038/s41586-021-03778-8, 34349261 PMC8756380

[ref172] World Health Organization (2025). Dementia. Available online at: https://www.who.int/zh/news-room/fact-sheets/detail/dementia (Accessed December 18 2025).

[ref173] Wu Y. Chen Y. Yu X. Zhang M. Li Z. (2023). Towards understanding neurodegenerative diseases: insights from *Caenorhabditis elegans*. Int. J. Mol. Sci. 25:443. doi: 10.3390/ijms25010443, 38203614 PMC10778690

[ref174] Wu Y. Tang W. Ding Y. Zhao Y. Zhou C. Cheng Y. . (2025). *Enterococcus faecalis* SI-FC-01 enhances the stress resistance and healthspan of *C. elegans* via AKT signaling pathway. Sci. Rep. 15:14454. doi: 10.1038/s41598-025-98440-y, 40281021 PMC12032096

[ref175] Wu J. You X. Cao Y. Xi J. Chen X. Zhang X. . (2025). High-throughput neurotoxicity study of neonicotinoids in *C. elegans*: oxidative stress and serotonergic neuronal damage as key mechanisms. Environ. Pollut. 383:126814. doi: 10.1016/j.envpol.2025.126814, 40651652

[ref176] Xie Q. Li K. Chen Y. Li Y. Jiang W. Cao W. . (2025). Gene therapy breakthroughs in ALS: a beacon of hope for 20% of ALS patients. Transl Neurodegener 14:19. doi: 10.1186/s40035-025-00477-6, 40234983 PMC12001736

[ref177] Xu R. Kang Q. Yang X. Yi P. Zhang R. (2025). Unraveling molecular targets for neurodegenerative diseases through *Caenorhabditis elegans* models. Int. J. Mol. Sci. 26:3030. doi: 10.3390/ijms26073030, 40243699 PMC11988803

[ref178] Xu Y. Taru H. Jin Y. Quinn C. C. (2015). SYD-1C, UNC-40 (DCC) and SAX-3 (Robo) function interdependently to promote axon guidance by regulating the MIG-2 GTPase. PLoS Genet. 11:e1005185. doi: 10.1371/journal.pgen.1005185, 25876065 PMC4398414

[ref179] Ye H. Robak L. A. Yu M. Cykowski M. Shulman J. M. (2023). Genetics and pathogenesis of Parkinson's syndrome. Annu. Rev. Pathol. 18, 95–121. doi: 10.1146/annurev-pathmechdis-031521-034145, 36100231 PMC10290758

[ref180] Yemini E. Lin A. Nejatbakhsh A. Varol E. Sun R. Mena G. E. . (2021). NeuroPAL: a multicolor atlas for whole-brain neuronal identification in *C. elegans*. Cell 184, 272–288.e11. doi: 10.1016/j.cell.2020.12.012, 33378642 PMC10494711

[ref181] Yogev S. Shen K. (2014). Cellular and molecular mechanisms of synaptic specificity. Annu. Rev. Cell Dev. Biol. 30, 417–437. doi: 10.1146/annurev-cellbio-100913-012953, 25150010

[ref182] Yue Z. Li Y. Yu B. Xu Y. Chen L. Chitturi J. . (2024). A leak K(+) channel TWK-40 sustains the rhythmic motor program. PNAS Nexus 3:pgae234. doi: 10.1093/pnasnexus/pgae234, 38957449 PMC11217676

[ref183] Zhang S. Li F. Zhou T. Wang G. Li Z. (2020). *Caenorhabditis elegans* as a useful model for studying aging mutations. Front. Endocrinol. (Lausanne) 11:554994. doi: 10.3389/fendo.2020.554994, 33123086 PMC7570440

[ref184] Zhao J. Ardiel E. Nurrish S. Kaplan J. M. (2026). Transsynaptic linking of calcium channels and postsynaptic receptors at a dyadic synapse. Proc. Natl. Acad. Sci. USA 123:e2603452123. doi: 10.1073/pnas.2603452123, 42018404 PMC13123913

[ref185] Zhao H. Shi G. Qin R. Sun Y. Guo W. Shi R. . (2026). Hungry for knowledge: Octopamine signaling regulates hunger-enhanced olfactory learning. Adv Sci (Weinh) 13:e13842. doi: 10.1002/advs.202513842, 41392936 PMC12948213

[ref186] Zheng S. Chiu H. Boudreau J. Papanicolaou T. Bendena W. Chin-Sang I. (2018). A functional study of all 40 *Caenorhabditis elegans* insulin-like peptides. J. Biol. Chem. 293, 16912–16922. doi: 10.1074/jbc.RA118.004542, 30206121 PMC6204898

[ref187] Zheng Q. Wang X. (2025). Alzheimer's disease: insights into pathology, molecular mechanisms, and therapy. Protein Cell 16, 83–120. doi: 10.1093/procel/pwae026, 38733347 PMC11786724

[ref188] Zhou X. Gueydan M. Jospin M. Ji T. Valfort A. Pinan-Lucarré B. . (2020). The netrin receptor UNC-40/DCC assembles a postsynaptic scaffold and sets the synaptic content of GABA(a) receptors. Nat. Commun. 11:2674. doi: 10.1038/s41467-020-16473-5, 32471987 PMC7260190

[ref189] Zhu R. Chin-Sang I. D. (2024). *C. elegans* insulin-like peptides. Mol. Cell. Endocrinol. 585:112173. doi: 10.1016/j.mce.2024.112173, 38346555

